# A historical perspective of biomedical explainable AI research

**DOI:** 10.1016/j.patter.2023.100830

**Published:** 2023-09-08

**Authors:** Luca Malinverno, Vesna Barros, Francesco Ghisoni, Giovanni Visonà, Roman Kern, Philip J. Nickel, Barbara Elvira Ventura, Ilija Šimić, Sarah Stryeck, Francesca Manni, Cesar Ferri, Claire Jean-Quartier, Laura Genga, Gabriele Schweikert, Mario Lovrić, Michal Rosen-Zvi

**Affiliations:** 1 Porini SRL, Via Cavour, 222074 Lomazzo, Italy; 2 AI for Accelerated Healthcare & Life Sciences Discovery, IBM R&D Laboratories, University of Haifa Campus, Mount Carmel, Haifa 3498825, Israel; 3 The Hebrew University of Jerusalem, Ein Kerem Campus, 9112102, Jerusalem, Israel; 4 Empirical Inference, Max-Planck Institute for Intelligent Systems, 72076 Tübingen, Germany; 5 Institute of Interactive Systems and Data Science, Graz University of Technology, Sandgasse 36/III, 8010 Graz, Austria; 6 Know-Center GmbH, Sandgasse 36/4A 8010, Graz, Austria; 7 Eindhoven University of Technology, 5135600 MB Eindhoven, The Netherlands; 8 Research Center Pharmaceutical Engineering GmbH, Inffeldgasse 138010 Graz, Austria; 9 Philips Research, HTC 4, 5656 AE Eindhoven, The Netherlands; 10 VRAIN, Universitat Politècnica de València, Camino de Vera, s/n 46022 Valencia, Spain; 11 Research Data Management, Graz University of Technology, Brockmanngasse 84, 8010 Graz, Austria; 12 School of Life Sciences, University of Dundee, Dow Street, Dundee DD1 5EH, UK; 13 Centre for Applied Bioanthropology, Institute for Anthropological Research, 10000 Zagreb, Croatia

**Keywords:** explainability, COVID-19, coronavirus, artificial intelligence, machine learning, meta-review, PRISMA, decision-making, trustworthiness, foundation models

## Abstract

The black-box nature of most artificial intelligence (AI) models encourages the development of explainability methods to engender trust into the AI decision-making process. Such methods can be broadly categorized into two main types: post hoc explanations and inherently interpretable algorithms. We aimed at analyzing the possible associations between COVID-19 and the push of explainable AI (XAI) to the forefront of biomedical research. We automatically extracted from the PubMed database biomedical XAI studies related to concepts of causality or explainability and manually labeled 1,603 papers with respect to XAI categories. To compare the trends pre- and post-COVID-19, we fit a change point detection model and evaluated significant changes in publication rates. We show that the advent of COVID-19 in the beginning of 2020 could be the driving factor behind an increased focus concerning XAI, playing a crucial role in accelerating an already evolving trend. Finally, we present a discussion with future societal use and impact of XAI technologies and potential future directions for those who pursue fostering clinical trust with interpretable machine learning models.

## The bigger picture

Understanding the inner working of machine-learning models has become a crucial point of discussion in fairness and reliability of artificial intelligence (AI). In this perspective, we reveal insights from recently published scientific works on explainable AI (XAI) within the biomedical sciences. Specifically, we speculate that the COVID-19 pandemic is associated with the rate of publications in the field. Current research efforts seem to be directed more toward explaining black-box machine-learning models than designing novel interpretable architecture. Notably, an inflection period in the publication rate was observed in October 2020, when the quantity of XAI research in biomedical sciences surged upward significantly.

While a universally accepted definition of explainability is unlikely, ongoing research efforts are pushing the biomedical field toward improving the robustness and reliability of applied machine learning, which we consider a positive trend.

## Graphical abstract

**Figure:**
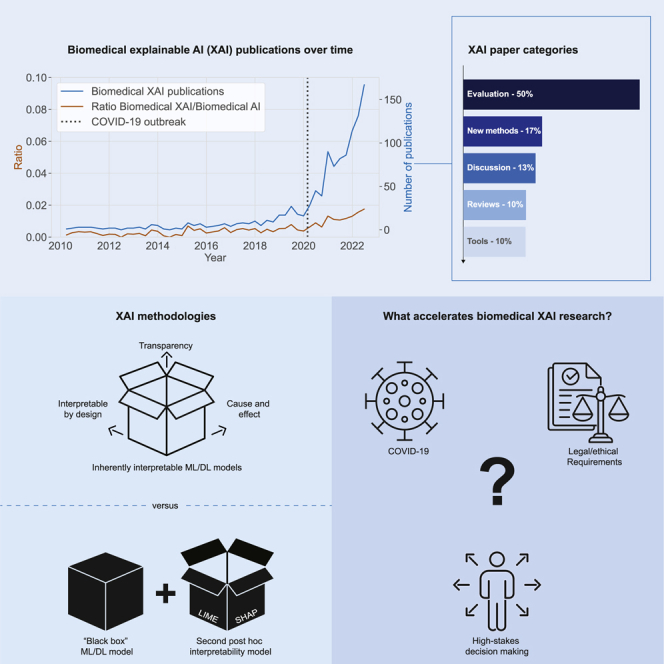

## Teaser

The field of explainable artificial intelligence (XAI) saw a rapid acceleration in the last few years that is contributing to discussions regarding requirements and definitions of interpretability. This perspective is the first to systematically analyze the trends in XAI research within the biomedical field considering the COVID-19 pandemic as a possible turning point. We explore the motivation of recent studies and highlight the contrast between interpretable models versus post hoc explanation and the debates regarding performance-interpretability tradeoff.

## Introduction

The COVID-19 pandemic has accelerated medical research and the way medical care is provided. Vaccines were developed and approved in record time,^1^ and novel drugs were rapidly designed for the treatment of severe acute respiratory syndrome coronavirus 2 (SARS-CoV-2) infection.^2^ Simultaneously, COVID-19 has caused major disruptions and backlogs in health systems, leaving many millions of people without care in the EU^3^ and around the world.^4^ As a direct response to the mounting pressures, primary and community care underwent a significant transformation, accelerating the use of remote consultation,^5^ automatic triage,^6^ and virtual monitoring and care.^7^^,^^8^ In order to decrease the backlog and restore and improve equitable healthcare, a combination of strategies have been identified, including the efficient adaptation of new technologies and digital solutions, additional funding, sufficient workforce, and infrastructure improvements.^3^ Even pre-COVID-19, information technology was suggested to be a key driver for higher quality, better effectiveness, and more efficiency in health systems,^9^ and several studies have found that computational models can be on par with or outperform human experts in certain diagnostic and prognostic tasks.^10^ However, while artificial intelligence (AI) technologies have become a growth engine in many other industries, development and adoption of such technologies across the health ecosystem have been much slower.^11^ During the pandemic, many AI research teams have stepped up their efforts in this application area, and hundreds of predictive tools to combat COVID-19 have been devised and, in some cases, applied in the clinic. Their potential and overall clinical relevancy are currently under critical discussion in several recent reviews.^12^^,^^13^^,^^14^ The opacity and black-box nature of many state-of-the-art AI systems additionally raise concerns in clinicians, care providers, and regulators and prevent their rapid integration into the high-stakes clinical decision-making process.^15^

It has been argued that explainable AI can engender transparency and increase adoption; however, the term “explainable AI” (XAI) is still not a well-defined concept. As discussed in Miller,^16^ explainability or interpretability is how human comprehensible the decisions of an AI system are, which is commonly referred to as XAI. The concept of XAI dates back to the mid-1970s, while the term itself is newer. It was coined in 2004, but only since 2016 has XAI received significant attention,^17^ including discussions of what its underlying concept is,^18^ the role of XAI in trustworthiness,^17^^,^^19^^,^^20^ and its importance in the biomedical domain.^21^^,^^22^

In recent years, a considerable number of review and perspective papers have been published with the goal of organizing the field of XAI (see, e.g., Adadi and Berrada,^17^ Barredo Arrieta et al.,^23^ Das and Rad,^24^ and Vilone and Longo,^25^ and references therein). Yet, only a few studies have focused on XAI in the field of medicine and health (see, e.g., Jiménez-Luna et al.,^26^ Tjoa and Guan,^27^ and Loh et al.^28^). Conversely, we have not found a study that focuses on how the field of biomedical XAI has changed in recent years. In this perspective, we provide a historical and conceptual perspective of the evolution of XAI research within biomedical sciences. We aim at giving an overview of the state-of-the-art XAI, the trends observed in the last few years, and the current explainability techniques. We hypothesize that the interest of the community is not primarily in the development of new XAI methods but rather in applying existent techniques to explain their black-box models. We hope that this work benefits researchers from various fields, serving as a reference point for those who want to get a deeper understanding of the direction in which XAI is going. The perspective is organized as follows: it starts with a discussion of the scope of XAI in the context of biomedicine and health. This is followed by a historical perspective, a review of the change in the extent of research performed over the years, and a conceptual perspective, a mapping of the research into five different types of studies. Finally, the perspective ends with emerging themes and open research areas in the field.

## Biomedical XAI: Overview of meaning and scope

The ultimate aim of XAI is to explain why a model produced a specific result. Such a goal can be achieved in a variety of manners, ranging from post hoc explanations to inductive biases that constrain model architectures. A particularly compelling framework to consider within this setting is the study of causality, through which we can move from observational explanations to a more in-depth analysis of the model in question, in the form of interventional and counterfactual interrogation. When a causal model is available, not only can we attempt to diagnose the working of the model, but we can also ask questions such as “how would this prediction change if a specific variable had a different value?” As a stricter framework, causality offers compelling tools to explain the workings of machine-learning models, and as such, we opted to include publications relevant to causality in biomedicine in the present meta-analysis. Undoubtedly, counterfactuals have become an essential part of XAI, enabling researchers to explain past outcomes and predict future events by identifying causal relations in the data.^29^^,^^30^ Thus, causal machine learning plays a central role in the biomedical domain.^31^^,^^32^ For instance, learning the optimal medication or therapy for a patient requires carefully curated data or causal effect estimation, which classic machine-learning models do not necessarily identify.^33^ Building causal models of the world can support the understanding of why a model selects a certain intervention as an optimal one, which in turn can help the AI developer in improving the technology, ultimately leading to an expansion of the medical knowledge. Notably, while XAI helps build user trust in the AI systems and provides useful insights for AI developers, trustworthy AI is a different concept. Trustworthiness encompasses not only technologies but also practices, and it has been institutionalized in policy documents guiding AI innovation, such as the EU’s High Level Expert Group on AI. To develop a historical view and a perspective on biomedical XAI, we started by reviewing citations from PubMed that fit the above scope definition.

PubMed is a free database of citations of publications in the biomedical domain that was developed by the National Center for Biotechnology Information (NCBI). It is a major source of literature analysis for biomedical researchers due to its comprehensiveness and accessibility. We performed a systematic review of PubMed papers following the recommendations of the PRISMA statement.^34^ We applied manual and automatic methods as illustrated in Figure S1; that is, we searched in the PubMed database for all citations until November 3, 2022, with no restrictions on regions, languages, or publication types. Citations were added to our cohort if they contained biomedical concepts in the abstracts or titles, which were automatically extracted based on a prespecified set of keywords (Table S1; Data S1). We excluded studies that were not related to AI and limited the search space with a start date of January 1, 2010 (defined based on the step change of significant attention to the advances of deep-learning technologies). Lastly, we included only papers that were related to XAI, considered as the wide term that includes concepts of the two broad and overlapping subjects discussed above: explainability and causal analysis. The complete information regarding the citation collection criteria used in the data collection process is illustrated in Figure S2. Manual review of the abstracts complemented the automatic extraction. The result is a cohort of 1,276 peer-reviewed papers that study biomedical XAI. In the next section, we discuss research trends that we found in our analysis.

## Biomedical XAI: A historical perspective through the lens of COVID-19

To investigate the trend of biomedical XAI and the possible role of the COVID-19 pandemic in bringing this field to the forefront of AI research, we used the publication date and constructed a time series defined by the number of biomedical XAI PubMed papers published per month. We focused on the date of COVID-19 outbreak, which was considered March 1, 2020. 261 abstracts on biomedical XAI were published before the outbreak and 1,017 after. After careful selection of studies and iterative manual revision of abstracts, we investigated whether publications related to causality and explainability showed the same trends pre- and post-COVID-19 (Figure S3). There was a much stronger growth in the rate of explainability publications than in causality, which could possibly be due to the difficulties in performing causal inference versus how convenient explaining an existing model is.

**Figure 1:**
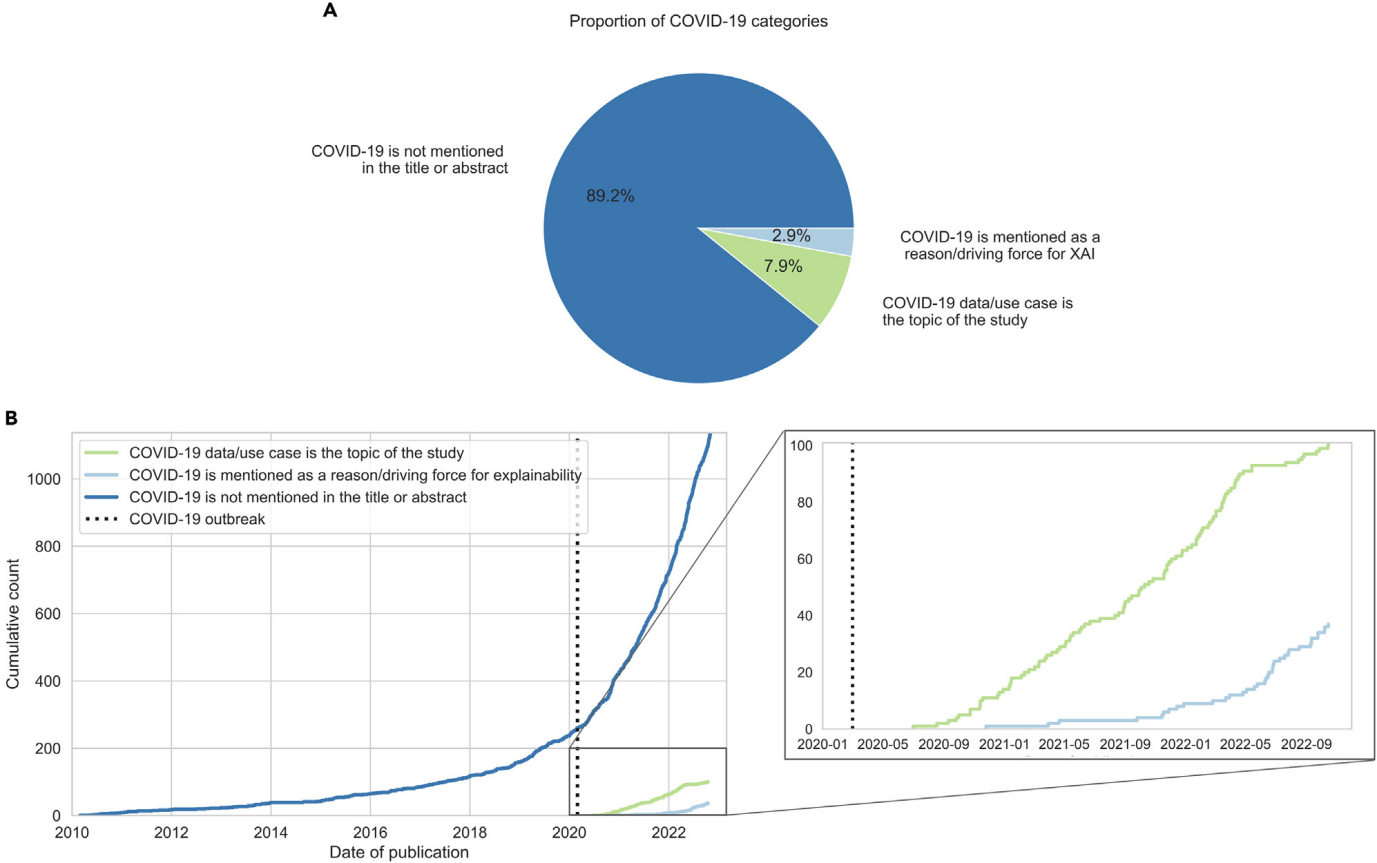
Trends in XAI papers related to COVID-19 (A) Proportions of COVID-19 categories in the dataset. (B) Cumulative number of XAI papers for each category throughout time. The increase in COVID-19-related papers, represented by the light blue and light green curves, set off around 7 months after the pandemic onset.

We then manually annotated the role of COVID-19 in each paper, where each annotator had three options to label a study: (1) studies that do not mention the pandemic in their titles or abstracts, (2) studies that used COVID-19 data (e.g., chest radiographs or computed tomography [CT] scans, number of SARS-CoV-2 infections, or administrated vaccines) to predict clinical outcomes, or (3) studies that mentioned COVID-19 as a reason or driving force for their research. Expectedly, the distribution of papers per label was uneven (Figure 1A): 1,137 studies (approximately 90%) did not mention the pandemic, whereas 101 studies (8%) used COVID-19 data and 37 studies mentioned COVID-19 as a driving force for their research. Interestingly, COVID-19-related publications significantly increased several months after the pandemic outbreak (Figure 1B, zoomed-in image), possibly representing the time period for researchers to collect enough data, design the studies, and start publishing them.

As an exploratory analysis, we investigated whether a change point is detected at the time of the pandemic outbreak or in the following months (Data S2). We found an inflection period in October 2020, when the quantity of XAI research in biomedical sciences surged upward significantly (Figure S4A). Pinpointing a specific cause for this acceleration is far from trivial. In the past 10 years, XAI has received increased attention in the form of funding, research programs, and legal and ethical requirements. Examples include the DARPA program^35^ or the European Commission’s White Paper on AI.^36^ However, we speculate that the advent of the COVID-19 pandemic itself could have been an important factor in bringing the field of XAI to the forefront in many AI-related research fields. The well-documented difficulties of AI (lack of impact of AI) in COVID-19 analysis could have motivated the need to better understand how models work and the origin of predictions.^12^^,^^13^ It is worth noting that across the most cited papers we reviewed, often the leading author (last co-author) was an AI researcher that expanded their horizon to the applied biomedical field.^37^^,^^38^

We then attempted to quantify the change observed and estimated that the field of biomedical XAI was pushed forward by about 25 months (Figure S4B). However, establishing the causal nature of this link would require a large-scale analysis of multiple research topics trends, which is beyond the scope of the current meta-analysis.

**Figure 2:**
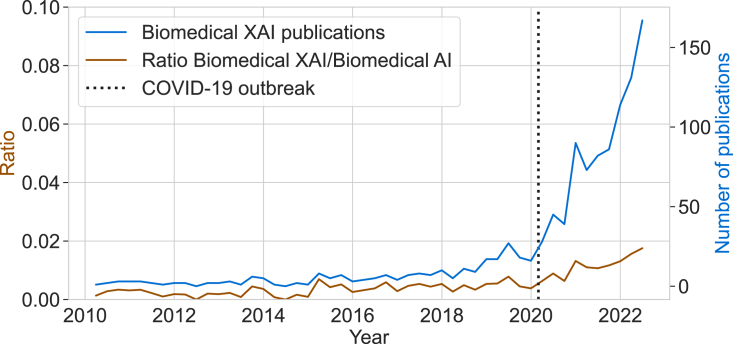
Comparison between the overall number of biomedical XAI publications (blue curve) and its proportion within the biomedical AI field (brown curve) Both plots show quarterly patterns of publications throughout time smoothed by a 1-year rolling average window.

Finally, we compared the numbers of biomedical XAI publications to the ratio between biomedical XAI and biomedical AI publications a long time (Figure 2). We found an exponential growth of biomedical XAI papers, which is followed by an increase in the ratio of biomedical XAI/biomedical AI papers. This is a faster exponential growth than the one detected in publications of AI in biomedical sciences in previous studies,^39^ as reflected by the constant increase in the ratio of biomedical XAI/biomedical AI papers. Importantly, the rapid increase in XAI publications is not only due to the increase in scientific literature using COVID-19 data (see, for instance, the exponential growth of the use of medical imaging and AI in the context of COVID-19^40^). On the contrary, our manual review showed that only 8% of the biomedical XAI papers were devoted to analysis of COVID-19 data, whereas 3% (37 papers) referred to COVID-19 as a motivation to discuss or apply XAI methods. This further corroborates our hypothesis that the advent of the COVID-19 pandemic acted as a catalyst and contributed to pushing the level of interest in XAI methodologies to a critical point in an already evolving trend.

The papers list, the manual annotation, and the code used for all analysis can be found in Fghisoni and Visonà.^41^

## Biomedical XAI: Study types

In order to get a more comprehensive view of the type of research performed in biomedical XAI, each abstract was annotated according to the type of study reported in the paper. We followed the approach discussed in Vilone and Longo^25^ and labeled the abstracts with one of the following categories that best described the study: (1) review or meta-analysis, (2) discussion of XAI concepts, (3) introduction of novel XAI methods, or (4) evaluation or application of XAI methods and added a fifth category, (5) datasets or tools that support XAI.

**Figure 3:**
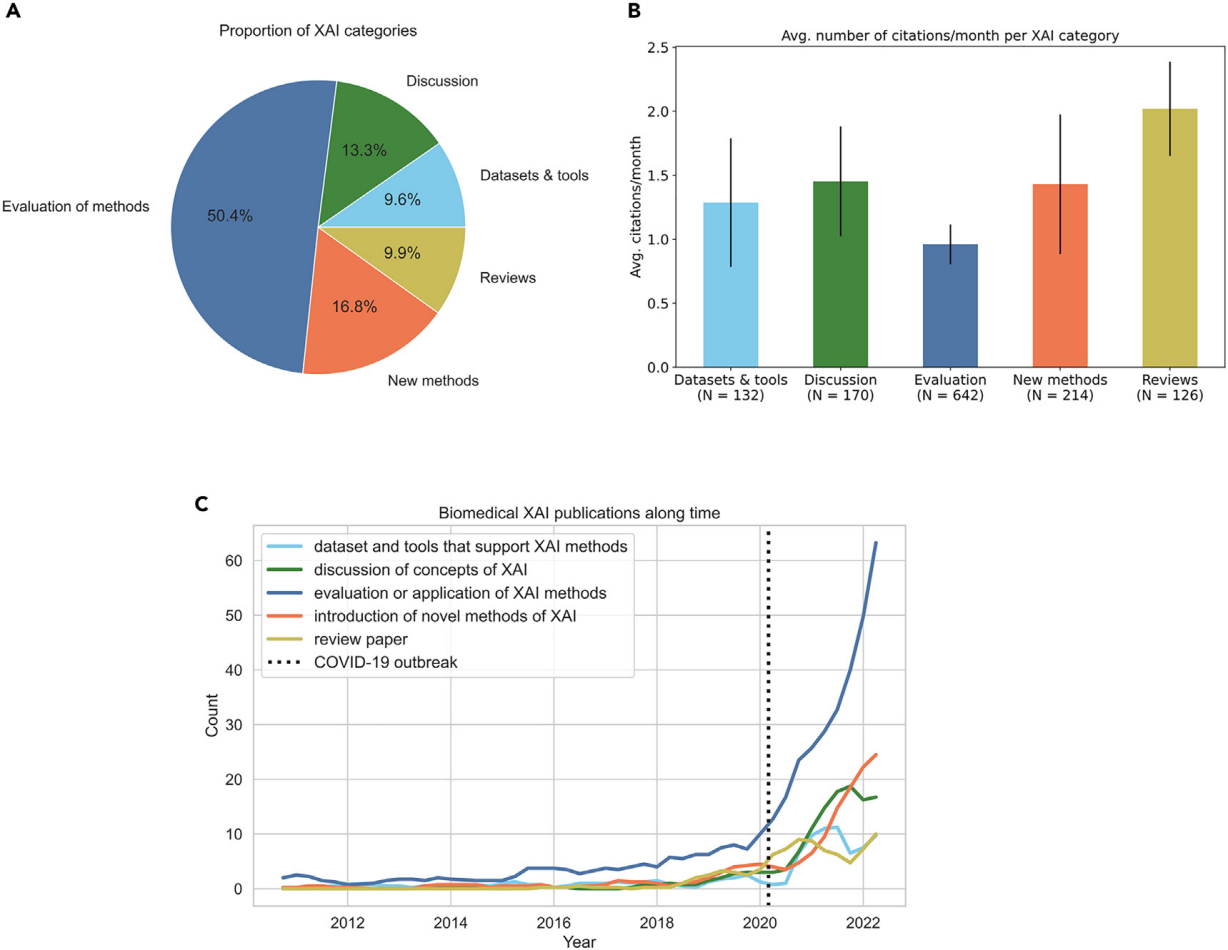
Biomedical XAI statistics and trends of the cohort of eligible papers (A) Proportions of biomedical XAI categories. (B) Average number of citations per month since published date. Error bars represent 95% confidence intervals. (C) Counts of biomedical XAI papers published per quarter year. For each category (colored curve), we smoothed quarterly patterns by using the 1 year rolling average window.

In total, 642 papers (approximately 50% of the eligible studies) evaluated or applied existing XAI methods in their respective studies, followed by 214 papers (17%) that introduced a novel XAI technology (either by innovating the way they explain their AI algorithms’ decisions or by adapting previous XAI ideas to a new research context; Figure 3A). Letters, comments, and narratives discussing XAI technologies comprised 13% of the cohort. Datasets or tools that support XAI technologies (e.g., software packages, AI frameworks and workflows, medical image datasets) and reviews on XAI methods composed together approximately 20% of the entire cohort.

We then tracked the Google Scholar citations of all eligible publications, considered as a proxy for public interest, and computed the number of citations per month since the date of publication (Figure 3B). As expected, review papers and novel methods attract most attention, as reflected by average citations per month, whereas application and evaluation of methods draw less attention. The relatively high average number of citations of papers with conceptual discussion is somewhat surprising and possibly reflects the heated debate on the role of XAI and its importance in the biomedical domain.

## Biomedical XAI: Future prospects

The debate around XAI is an ongoing effort that requires input from experts in computer science, philosophy, ethics, and law. The largest challenges arise from the difficulty of defining and quantifying “explainability”: how do we decide if the output of a model is interpretable? A very deep decision tree model, for example, would offer a direct explanation for the predicted outcome while being useless for human interpretation due to the sheer amount of split choices presented. And yet, despite these obstacles, ongoing discussion is necessary for the advancement of AI, especially in high-stakes settings, not only to improve robustness and reliability but also to satisfy the growing levels of legal requirements for the “right of explanation” that is being adopted to protect the stakeholders involved.^42^

Briefly, XAI algorithms can be divided into two main categories: transparent models that convey some intrinsic degree of interpretability and post hoc explainability techniques. One of the most prominent directions in the field is progressing toward leveraging the latter techniques, where explanation is provided to existing opaque models rather than the *de novo* design of methods rooted in causality. When analyzing the content of the ten most popular papers (based on their average citations), it was observed that Lundberg et al.’s study^43^ was considerably the most cited one, averaging over 60 citations per month since its publication. Among these papers, four of them,^43^^,^^44^^,^^45^^,^^46^ including Lundberg and colleagues’ work, present post hoc explainability methods, while the remaining six papers^47^^,^^48^^,^^49^^,^^50^^,^^51^^,^^52^ focus on developing inherently interpretable models. This tendency to explore post hoc solution could stem from the fact that several explanation techniques can be applied to a variety of models with minimal added efforts. Lundberg’s Shapley additive explanations (SHAP) analysis was frequently used in the biomedical literature that we reviewed. Although the majority of current post hoc methods only provide locally reliable explanations and can be misleading of model functionality,^53^ Shapley values could be the sole approach that complies with legal requirements due to its foundation in a solid theory derived from axioms of a fair game.^54^

Above these variety of approaches, there is an ongoing debate regarding the tradeoff between performance and interpretability of machine-learning models. Widespread in the literature is the opinion that retaining or designing interpretability into an ML solution comes at a cost in accuracy of the predictions. It has been argued that such a tradeoff is but a myth,^55^ and to an extent this holds true. Often, different models, including explainable ones, offer very similar performances.^56^ Within these settings, it is therefore obvious to choose the solution that is also easier to interpret.

However, recent advances in large-scale deep-learning models have challenged this idea once again. The successes of foundation models,^57^ which are those models trained on broad data and then adapted to specific tasks, have put in question whether any explainable model could reach the same level of performance. The results produced by BERT,^58^ GPT-3,^59^ and DALL-E^60^ are outclassing those of any transparent model by a wide margin. Recently, it was shown that large language models such as ChatGPT^61^ may have the potential to assist with clinical reasoning.^62^ Hence, it might become a tool to explain AI-based approaches and support AI-based decisions.

The paradigm shift produced by foundation models can offer completely different opportunities to make use of interpretations of their results. While a common approach with simpler models would be to diagnose the elements that lead to specific predictions (e.g., analyze the importance of features), with large-scale models, it is possible to obtain novel designs derived from the surprising emergent properties learned from the sheer quantity of data, which could expand our understanding of the task at hand in completely unexpected directions. Within the biomedical field, this could lead to, for example, the design of novel proteins that are not observed in nature^63^ or to exploring new connections between diseases.^64^

Are the post hoc explainable technologies satisfying? Does the success of large-scale deep-learning models mean that it is futile to pursue non-post hoc explainability in AI? Perhaps it represents an unreachable goal. However, the increase in BXAI publications, highlighted in our survey, indicates that many research groups believe that the future of AI-based advances in medicine and health belongs to those who successfully address such questions.

## Data and code availability

All data analyzed and produced in this study, as well as the original code, have been deposited at https://github.com/gvisona/BiomedXAI and at the repository in Zenodo (https://doi.org/10.5281/zenodo.8207487). Data are publicly available as of the date of publication.

## Supplemental information

Document S1. Data S1 and S2, Table S1, and Figures S1–S4

Document S2. Article plus supplemental information
