# Supplementary material for: A historical perspective of biomedical explainable AI research
Source: Patterns (N Y). 2023 Sep 8;4(9):100830. doi: 10.1016/j.patter.2023.100830 (PMC10500028; doi:10.1016/j.patter.2023.100830)
Supplement: Document S1. Data S1 and S2, Table S1, and Figures S1–S4 [file mmc1.pdf]

Patterns, Volume *4*

Supplemental information

A historical perspective

# of biomedical explainable AI research

Luca Malinverno, Vesna Barros, Francesco Ghisoni, Giovanni Visonà, Roman Kern, Philip J. Nickel, Barbara Elvira Ventura, Ilija Šimić, Sarah Stryeck, Francesca Manni, Cesar Ferri, Claire Jean-Quartier, Laura Genga, Gabriele Schweikert, Mario Lovrić, and Michal Rosen-Zvi

Data S1

Section 1: Search strategy

The search strategy in the PubMed database was performed on November 3rd, 2022, and the study flow is illustrated in Figure E1. First, we focused on the automatic extraction of biomedical papers. We consulted with the Physician Specialty Data Report of the Association of American Medical Colleges and selected as keywords of biomedical papers all specialities that have more than 10,000 active physicians (as of 2019). Second, we extracted papers related to XAI within the biomedical sciences. To do so, we focused on concepts of explainability and causal inference. Table E1 provides a summary of the different terminologies we used to access citations of these two groups. The search strategy had the following steps:

1. Explore the full space of all PubMed citations.
2. Extract all citations whose title or abstract refers to biomedical concepts (using the keywords "anesthesiology", "biomedical", "biomedicine", "bioinformatics", "cardiovascular", "clinical", "critical care", "dermatology", "disease", "drug","gastroenterology", "genetics", "genomics", "gynecology", "health", "healthcare", "hematology", "immunology", "life sciences" "medical", "medicine", "nephrology", "neurology", "obstetrics", "oncology","ophthalmology", "orthopedic","pathology", "pediatrics", "pharmacology", "protein", "psychiatry", "radiology", "surgery", "urology", "vaccine").
3. From the subset of biomedical cohort, extract all citations whose title or abstract refer to AI concepts (using the keywords "AI", "artificial intelligence", "deep learning" and "machine learning").
4. Limit the cohort to be composed of papers published since Jan 1, 2010.
5. From the subset above, extract papers related to explainability and/or causal (using the keywords listed in Table 1) while recording which of the three concepts (or combination of them) are used to extract the paper: explainability, causal or both.

Table S1: Keywords used to extract XAI-related abstracts and define the two cohorts: explainability and causal.

Terminology	Keywords	Explanation
Explainability	"black box models", "explainable", "interpretability", "explainability", "opacity", "opaque", “xai”	Interpretable and comprehensible models and truly explainable models offering an automated reasoning that explains the decision making process of a model using human-understandable features of the input data [1].
Causal	"causal", "causation", "counterfactual”	Finding out how much would the effect variable(s) change as a result of modifying the value of input variable(s) and discovering which variables could be modified in order to change the value of the effect variable(s)[2].

The inclusion/exclusion criteria resulted in the creation of the cohort exemplified in Figure E2. Among all PubMed citations retrieved on November 3rd, 2022 (over 34 million), we excluded the ones not related to biomedical concepts (n = 12·4 million) or AI (n = 21·4 million). Of 148,700 remaining papers, we excluded 27,851 that were published before 2010. This left 120,849 papers, from which 980 referred to explainability, 623 referred to causality and 14 papers that referred to both. We manually reviewed 1,603 abstracts belonging to these two groups with respect to language, category of the paper and the role of COVID-19 in the context of the studies, 1,276 of which met criteria for final inclusion.

When analysing the differences in trends between publications with "causal" terms versus publications with "explainability" terms, we found that they followed different growth trends, as shown in Figure E3.

Data S2

Section 1: Trend analysis

We assumed that the number of monthly biomedical XAI publications in general follows an exponential curve, a common assumption when modelling the growing number of publications in a field [3,4]. More specifically, we followed the ideas presented in [5] and modeled changing trends as a piecewise Negative Binomial Regression task. As the data available is counts over time, we deemed a Poisson-like regression method to be more appropriate than a simple exponential fit; due to detected overdispersion, we selected an NB1 Negative Binomial[6] rather than a simple Poisson model. The assumed functional form is:

$$\begin{gathered} y(x) \sim NB(\mu(x), \alpha) \\ \mu(x) = \beta_0 + \beta_1 * x + \beta_2 * \theta_D \\ Var[y|x] = \mu(x) + \alpha\mu(x) \end{gathered} \tag{1}$$

where y is the number of monthly biomedical XAI papers, x is the number of months elapsed since the start of the time series, a is a parameter to be fit and θ_D is a Heaviside step function that has value 0 for dates before the transition date D, and value one afterwards.

We define the task of detecting a change in trend as the determination of the date *D* where the threshold indicator θ_D should change value to best explain the observed data. To find this date, we examine the goodness of fit of Equation 1 for varying change points *D*. Afterwards, we compare the performance of this piecewise exponential fit to a single fitting function (equivalent to setting the parameters *b* and *d*) using an F-test.

After determining the changepoint *D*, we attempt to interpret it and quantify the observed effect by fitting a different Negative Binomial model, of the form:

$$y(x) \sim NB(\mu(x), \alpha)$$
$$\mu(x) = \beta_0 + \beta_1 * (x + \Delta * \theta_D) \quad (2)$$
$$Var[y|x] = \mu(x) + \alpha\mu(x)$$

Under the assumption that without external intervention the progression of a series should be smooth, this second fitting task is equivalent to asking: "When would we have likely observed the data points after D if the intervention never happened?". The trend changepoint analysis was performed with Python's *scipy* library (version 1·9·3; https://scipy.org) [7] and *statmodels* library (version 0.13.5; https://statsmodels.org) [8].

We fit the Negative Binominal Regression presented in Equation 1 for varying threshold months *D* and evaluated the goodness of the fit using the Coefficient of Determination R^2 and the Sum of Squared Errors (SSE, also called Residual Sum of Squares). The results, presented in the upper part of Figure E4, show that the optimal fit is achieved when modelling a change in trend starting from October 2020. The corresponding piecewise fit is shown in the middle portion of the figure.

We compared the piecewise function with a trend change in October 2020 against the single fitting function (with $\theta_D = 0$, or equivalently $\beta_2 = 0$) using an F-test. The resulting F-statistic of 40·9 results in a p-value of $1 * 10^{-63}$, which supports the claim that the improvement brought by the piecewise fit is statistically significant for a threshold of 0·01 even after adjustments for hypothesis testing.

Robustly defining the cause of this trend change is far from trivial, however we hypothesize that the outbreak of the COVID-19 pandemic is the main external contributor to affect an already growing trend. Considering the months necessary to gather and analyze data, and compile the results in a published paper, it is plausible to consider that the effect of the pandemic on the rate of publications would require several months to appear.

We then fixed the detected trend changepoint in October 2020, and fit the regression model in Equation 2, where the effect of the changepoint is modelled as a shift in time. The resulting fit, shown in Figure E4.B, is that the trend change is equivalent to a shift of 25 months of the points observed after October 2020. Under the assumption of smoothness in the absence of interventions, we would have therefore expected to see the rates of publications observed after October 2020 only after August 2022. Equivalently, we can describe the effect of the trend change as pushing forward the rate of publications in biomedical XAI by 25 months.

Supplemental references

1. Doran D, Schulz S, Besold TR. What Does Explainable AI Really Mean? A New Conceptualization of Perspectives [Internet]. arXiv; 2017 [cited 2022 Nov 4]. Available from: http://arxiv.org/abs/1710.00794

2. Guo R, Cheng L, Li J, Hahn PR, Liu H. A Survey of Learning Causality with Data: Problems and Methods. ACM Comput Surv. 2021 Jul 31;53(4):1–37.

3. Parolo PDB, Pan RK, Ghosh R, Huberman BA, Kaski K, Fortunato S. Attention decay in science. Journal of Informetrics. 2015 Oct 1;9(4):734–45.

4. Bornmann L, Mutz R. Growth rates of modern science: A bibliometric analysis based on the number of publications and cited references. Journal of the Association for Information Science and Technology. 2015;66(11):2215–22.

5. Bornmann L, Haunschild R, Mutz R. Growth rates of modern science: a latent piecewise growth curve approach to model publication numbers from established and new literature databases. Humanit Soc Sci Commun. 2021 Oct 7;8(1):1–15.

6. Greene W. Functional forms for the negative binomial model for count data. Economics Letters. 2008 Jun 1;99(3):585–90.

7. Virtanen P, Gommers R, Oliphant TE, Haberland M, Reddy T, Cournapeau D, et al. SciPy 1.0: fundamental algorithms for scientific computing in Python. Nat Methods. 2020 Mar;17(3):261–72.

8. Seabold S, Perktold J. Statsmodels: Econometric and Statistical Modeling with Python. In Austin, Texas; 2010 [cited 2023 Mar 1]. p. 92–6. Available from: https://conference.scipy.org/proceedings/scipy2010/seabold.html

Figure captions

Figure E1: Illustration of the study flow that started with a set of questions (yellow boxes), followed by data collection from PubMed (middle bottleneck) and a manual and automatic study (green boxes).

Figure E2: Flowchart with citation collection criteria used in the data collection process.

Figure E3: Cumulative count of publications related to causality and explainability.

Figure E4: Trend analysis. **A:** In the upper and lower portion of the figure, we displayed the goodness-of-fit measures SSE and R^2, respectively, which were obtained when varying the threshold date D for the indicator θ_D and fitting the piecewise NB1 Negative Binomial function from Equation 1. In the middle part, the piecewise fit corresponding to the best changepoint (October 2020). **B:** The Negative Binomial fit modelled by Equation 2. In light grey, the data points observed after October 2020; the corresponding points shifted by Δ months are shown in the right part of the plot. In both analyses, all parameters and their 95% confidence interval were estimated via bootstrapping (N=1000).

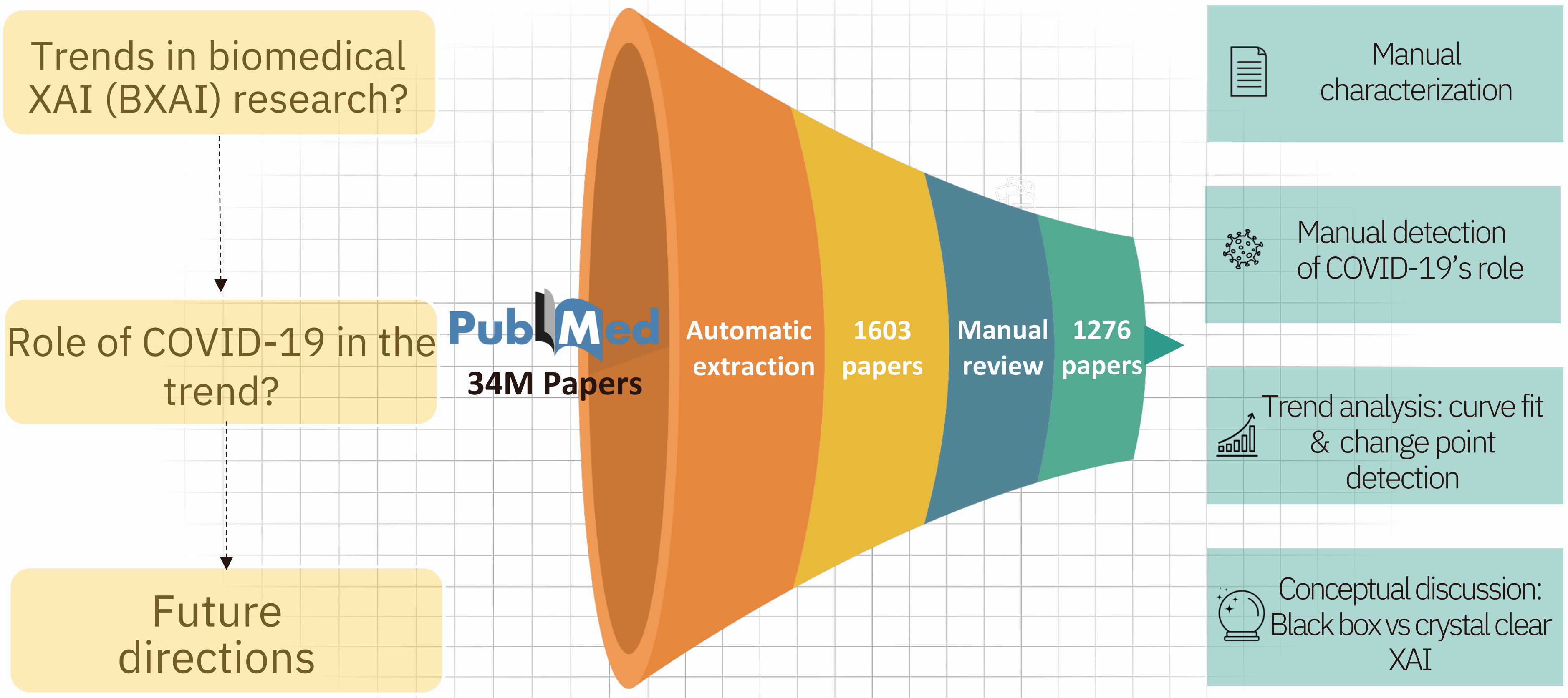

Trends in biomedical XAI (BXAI) research?
Role of COVID-19 in the trend?
Future directions
PubMed
34M Papers
Automatic extraction
1603 papers
Manual review
1276 papers
Manual characterization
Manual detection of COVID-19's role
Trend analysis: curve fit & change point detection
Conceptual discussion: Black box vs crystal clear XAI

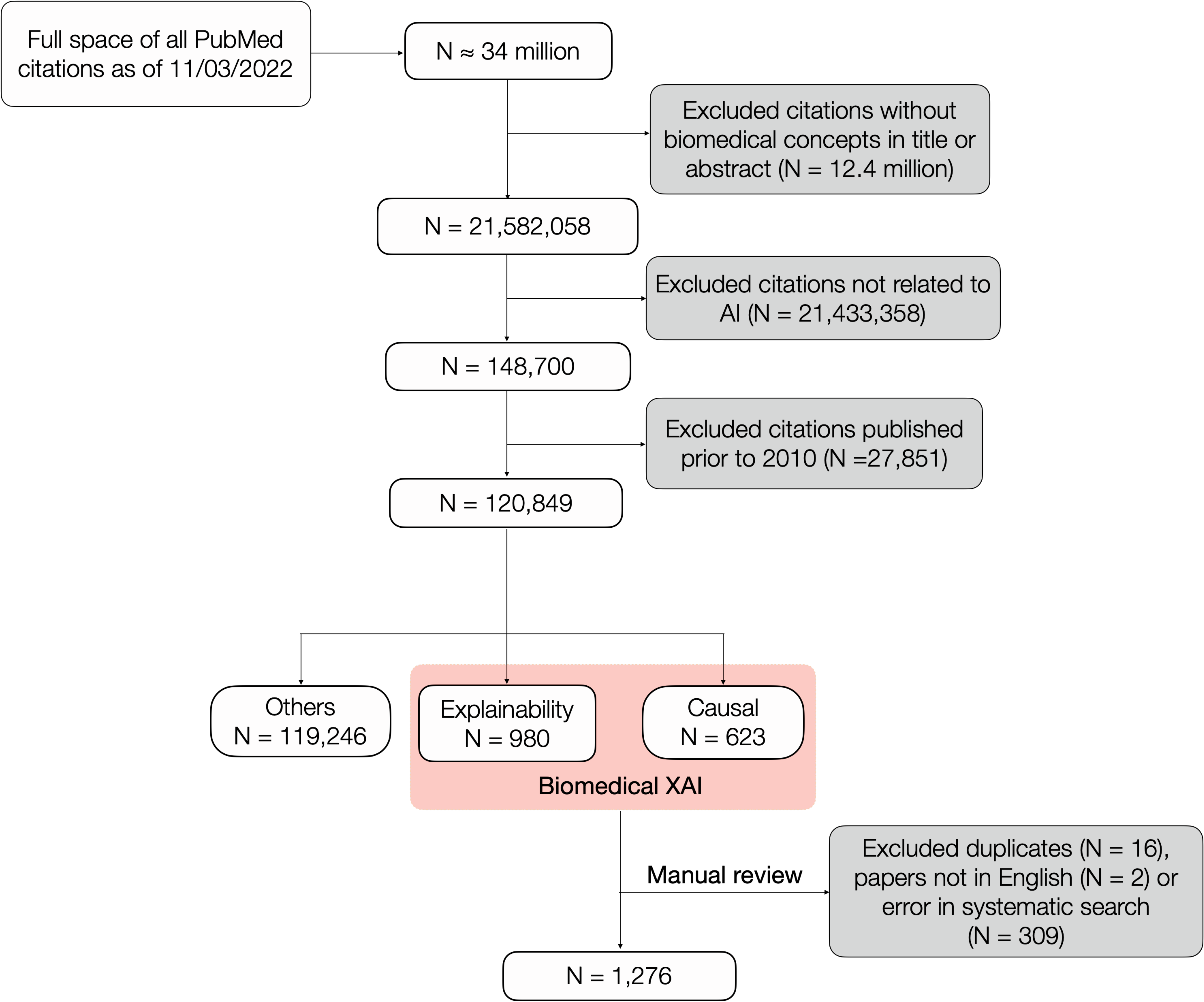
Full space of all PubMed citations as of 11/03/2022
N ≈ 34 million
Excluded citations without biomedical concepts in title or abstract (N = 12.4 million)
N = 21,582,058
Excluded citations not related to AI (N = 21,433,358)
N = 148,700
Excluded citations published prior to 2010 (N =27,851)
N = 120,849
Others N = 119,246
Explainability N = 980
Causal N = 623
Biomedical XAI
Manual review
Excluded duplicates (N = 16), papers not in English (N = 2) or error in systematic search (N = 309)
N = 1,276

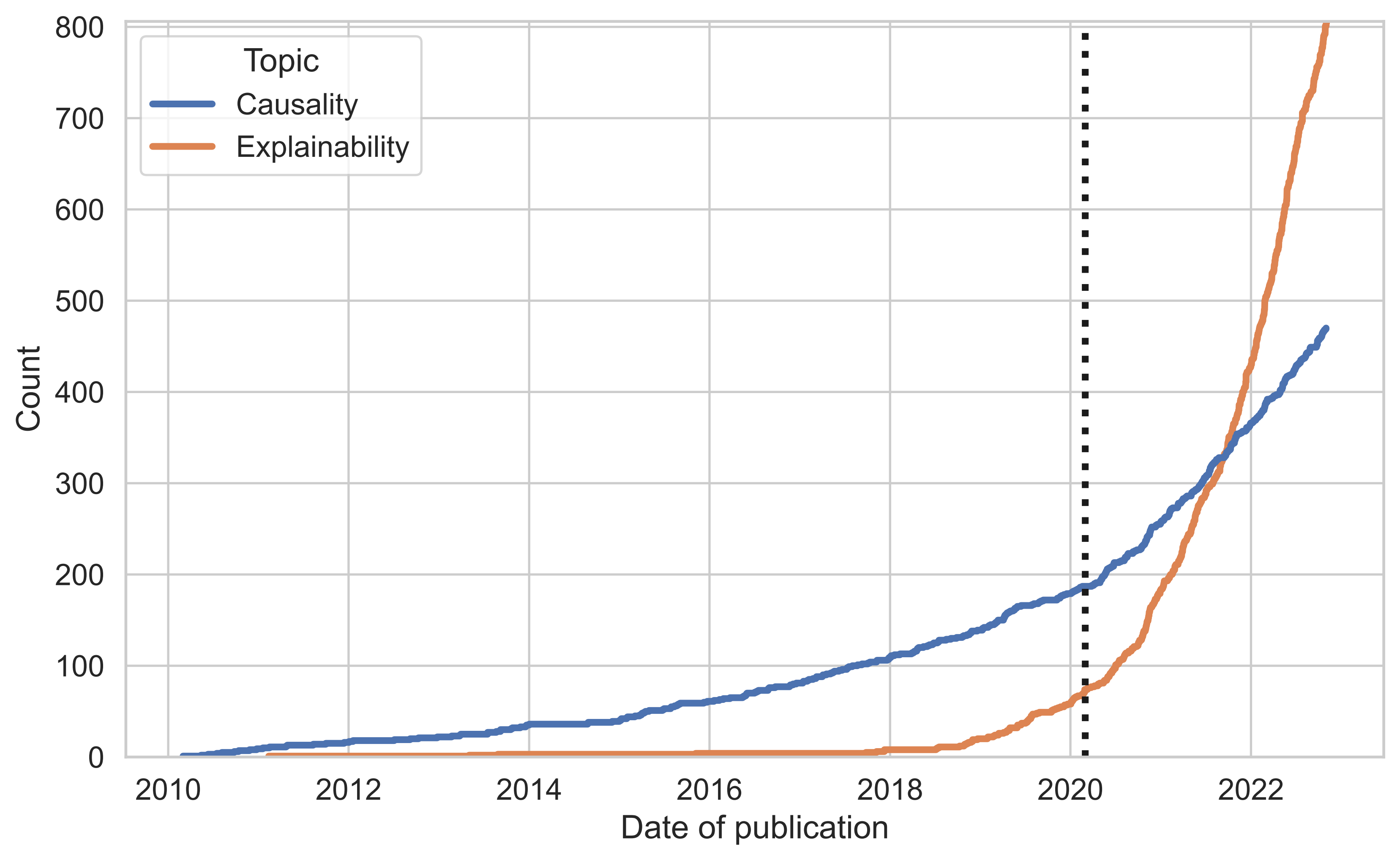

Topic
Causality
Explainability
Count
0
100
200
300
400
500
600
700
800
2010
2012
2014
2016
2018
2020
2022
Date of publication

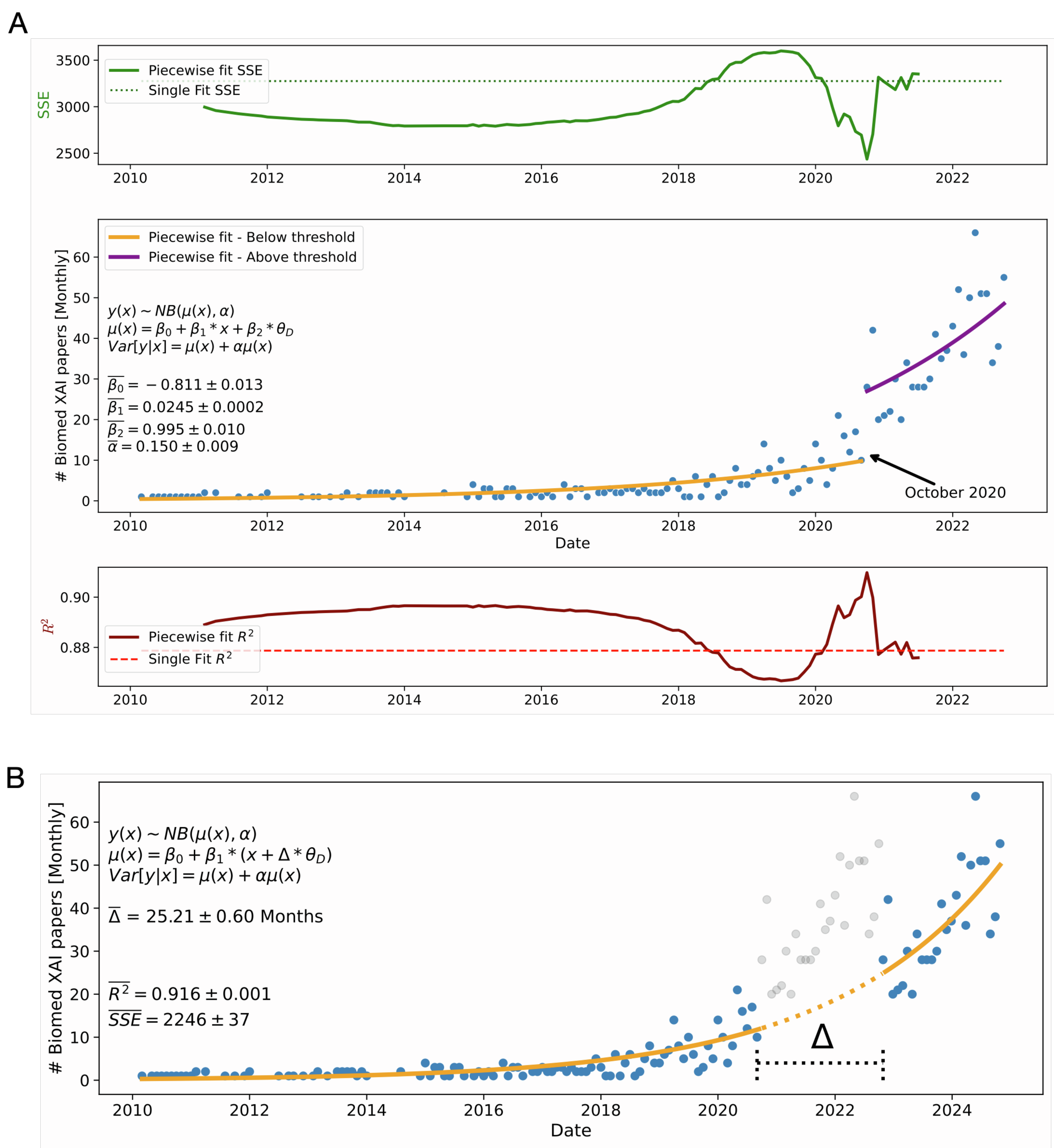
A
Piecewise fit SSE
Single Fit SSE
SSE
3500
3000
2500
2010
2012
2014
2016
2018
2020
2022
Piecewise fit - Below threshold
Piecewise fit - Above threshold
$y(x) \sim NB(\mu(x), \alpha)$
$\mu(x) = \beta_0 + \beta_1 * x + \beta_2 * \theta_D$
$Var[y|x] = \mu(x) + \alpha\mu(x)$
$\overline{\beta_0} = -0.811 \pm 0.013$
$\overline{\beta_1} = 0.0245 \pm 0.0002$
$\overline{\beta_2} = 0.995 \pm 0.010$
$\overline{\alpha} = 0.150 \pm 0.009$
Biomed XAI papers [Monthly]
October 2020
Date
R^2
0.90
0.88
Piecewise fit R^2
Single Fit R^2
B
$y(x) \sim NB(\mu(x), \alpha)$
$\mu(x) = \beta_0 + \beta_1 * (x + \Delta * \theta_D)$
$Var[y|x] = \mu(x) + \alpha\mu(x)$
$\overline{\Delta} = 25.21 \pm 0.60$ Months
$\overline{R^2} = 0.916 \pm 0.001$
$\overline{SSE} = 2246 \pm 37$
Δ
Biomed XAI papers [Monthly]
2024
Date
