# Supplementary material for: A historical perspective of biomedical explainable AI research
Source: Patterns (N Y). 2023 Sep 8;4(9):100830. doi: 10.1016/j.patter.2023.100830 (PMC10500028; doi:10.1016/j.patter.2023.100830)
Supplement: Document S2. Article plus supplemental information [file mmc2.pdf]

Patterns

CellPress
OPEN ACCESS

Perspective

# A historical perspective of biomedical explainable AI research

Luca Malinverno,[1,14] Vesna Barros,[2,3,14,*] Francesco Ghisoni,[1,14] Giovanni Visonà,[4] Roman Kern,[5,6] Philip J. Nickel,[7] Barbara Elvira Ventura,[1] Ilija Šimić,[6] Sarah Stryeck,[8] Francesca Manni,[9] Cesar Ferri,[10] Claire Jean-Quartier,[11] Laura Genga,[7] Gabriele Schweikert,[12] Mario Lovrić,[6,13] and Michal Rosen-Zvi[2,3]
[1]Porini SRL, Via Cavour, 222074 Lomazzo, Italy
[2]AI for Accelerated Healthcare & Life Sciences Discovery, IBM R&D Laboratories, University of Haifa Campus, Mount Carmel, Haifa 3498825, Israel
[3]The Hebrew University of Jerusalem, Ein Kerem Campus, 9112102, Jerusalem, Israel
[4]Empirical Inference, Max-Planck Institute for Intelligent Systems, 72076 Tübingen, Germany
[5]Institute of Interactive Systems and Data Science, Graz University of Technology, Sandgasse 36/III, 8010 Graz, Austria
[6]Know-Center GmbH, Sandgasse 36/4A 8010, Graz, Austria
[7]Eindhoven University of Technology, 5135600 MB Eindhoven, The Netherlands
[8]Research Center Pharmaceutical Engineering GmbH, Inffeldgasse 138010 Graz, Austria
[9]Philips Research, HTC 4, 5656 AE Eindhoven, The Netherlands
[10]VRAIN, Universitat Politècnica de València, Camino de Vera, s/n 46022 Valencia, Spain
[11]Research Data Management, Graz University of Technology, Brockmanngasse 84, 8010 Graz, Austria
[12]School of Life Sciences, University of Dundee, Dow Street, Dundee DD1 5EH, UK
[13]Centre for Applied Bioanthropology, Institute for Anthropological Research, 10000 Zagreb, Croatia
[14]These authors contributed equally
*Correspondence: vesna.barros@ibm.com
https://doi.org/10.1016/j.patter.2023.100830

**THE BIGGER PICTURE** Understanding the inner working of machine-learning models has become a crucial point of discussion in fairness and reliability of artificial intelligence (AI). In this perspective, we reveal insights from recently published scientific works on explainable AI (XAI) within the biomedical sciences. Specifically, we speculate that the COVID-19 pandemic is associated with the rate of publications in the field. Current research efforts seem to be directed more toward explaining black-box machine-learning models than designing novel interpretable architecture. Notably, an inflection period in the publication rate was observed in October 2020, when the quantity of XAI research in biomedical sciences surged upward significantly. While a universally accepted definition of explainability is unlikely, ongoing research efforts are pushing the biomedical field toward improving the robustness and reliability of applied machine learning, which we consider a positive trend.

1 **2** 3 4 5 **Proof-of-Concept:** Data science output has been formulated, implemented, and tested for one domain/problem

## SUMMARY

The black-box nature of most artificial intelligence (AI) models encourages the development of explainability methods to engender trust into the AI decision-making process. Such methods can be broadly categorized into two main types: post hoc explanations and inherently interpretable algorithms. We aimed at analyzing the possible associations between COVID-19 and the push of explainable AI (XAI) to the forefront of biomedical research. We automatically extracted from the PubMed database biomedical XAI studies related to concepts of causality or explainability and manually labeled 1,603 papers with respect to XAI categories. To compare the trends pre- and post-COVID-19, we fit a change point detection model and evaluated significant changes in publication rates. We show that the advent of COVID-19 in the beginning of 2020 could be the driving factor behind an increased focus concerning XAI, playing a crucial role in accelerating an already evolving trend. Finally, we present a discussion with future societal use and impact of XAI technologies and potential future directions for those who pursue fostering clinical trust with interpretable machine learning models.


This is an open access article under the CC BY license (http://creativecommons.org/licenses/by/4.0/).

## INTRODUCTION

The COVID-19 pandemic has accelerated medical research and the way medical care is provided. Vaccines were developed and approved in record time,[1] and novel drugs were rapidly designed for the treatment of severe acute respiratory syndrome coronavirus 2 (SARS-CoV-2) infection.[2] Simultaneously, COVID-19 has caused major disruptions and backlogs in health systems, leaving many millions of people without care in the EU[3] and around the world.[4] As a direct response to the mounting pressures, primary and community care underwent a significant transformation, accelerating the use of remote consultation,[5] automatic triage,[6] and virtual monitoring and care.[7,8] In order to decrease the backlog and restore and improve equitable healthcare, a combination of strategies have been identified, including the efficient adaptation of new technologies and digital solutions, additional funding, sufficient workforce, and infrastructure improvements.[3] Even pre-COVID-19, information technology was suggested to be a key driver for higher quality, better effectiveness, and more efficiency in health systems,[9] and several studies have found that computational models can be on par with or outperform human experts in certain diagnostic and prognostic tasks.[10] However, while artificial intelligence (AI) technologies have become a growth engine in many other industries, development and adoption of such technologies across the health ecosystem have been much slower.[11] During the pandemic, many AI research teams have stepped up their efforts in this application area, and hundreds of predictive tools to combat COVID-19 have been devised and, in some cases, applied in the clinic. Their potential and overall clinical relevancy are currently under critical discussion in several recent reviews.[12–14] The opacity and black-box nature of many state-of-the-art AI systems additionally raise concerns in clinicians, care providers, and regulators and prevent their rapid integration into the high-stakes clinical decision-making process.[15]

It has been argued that explainable AI can engender transparency and increase adoption; however, the term "explainable AI" (XAI) is still not a well-defined concept. As discussed in Miller,[16] explainability or interpretability is how human comprehensible the decisions of an AI system are, which is commonly referred to as XAI. The concept of XAI dates back to the mid-1970s, while the term itself is newer. It was coined in 2004, but only since 2016 has XAI received significant attention,[17] including discussions of what its underlying concept is,[18] the role of XAI in trustworthiness,[17,19,20] and its importance in the biomedical domain.[21,22]

In recent years, a considerable number of review and perspective papers have been published with the goal of organizing the field of XAI (see, e.g., Adadi and Berrada,[17] Barredo Arrieta et al.,[23] Das and Rad,[24] and Vilone and Longo,[25] and references therein). Yet, only a few studies have focused on XAI in the field of medicine and health (see, e.g., Jiménez-Luna et al.,[26] Tjoa and Guan,[27] and Loh et al.[28]). Conversely, we have not found a study that focuses on how the field of biomedical XAI has changed in recent years. In this perspective, we provide a historical and conceptual perspective of the evolution of XAI research within biomedical sciences. We aim at giving an overview of the state-of-the-art XAI, the trends observed in the last few years, and the current explainability techniques. We hypothesize that the interest of the community is not primarily in the development of new XAI methods but rather in applying existent techniques to explain their black-box models. We hope that this work benefits researchers from various fields, serving as a reference point for those who want to get a deeper understanding of the direction in which XAI is going. The perspective is organized as follows: it starts with a discussion of the scope of XAI in the context of biomedicine and health. This is followed by a historical perspective, a review of the change in the extent of research performed over the years, and a conceptual perspective, a mapping of the research into five different types of studies. Finally, the perspective ends with emerging themes and open research areas in the field.

## BIOMEDICAL XAI: OVERVIEW OF MEANING AND SCOPE

The ultimate aim of XAI is to explain why a model produced a specific result. Such a goal can be achieved in a variety of manners, ranging from post hoc explanations to inductive biases that constrain model architectures. A particularly compelling framework to consider within this setting is the study of causality, through which we can move from observational explanations to a more in-depth analysis of the model in question, in the form of interventional and counterfactual interrogation. When a causal model is available, not only can we attempt to diagnose the working of the model, but we can also ask questions such as "how would this prediction change if a specific variable had a different value?" As a stricter framework, causality offers compelling tools to explain the workings of machine-learning models, and as such, we opted to include publications relevant to causality in biomedicine in the present meta-analysis. Undoubtedly, counterfactuals have become an essential part of XAI, enabling researchers to explain past outcomes and predict future events by identifying causal relations in the data.[29,30] Thus, causal machine learning plays a central role in the biomedical domain.[31,32] For instance, learning the optimal medication or therapy for a patient requires carefully curated data or causal effect estimation, which classic machine-learning models do not necessarily identify.[33] Building causal models of the world can support the understanding of why a model selects a certain intervention as an optimal one, which in turn can help the AI developer in improving the technology, ultimately leading to an expansion of the medical knowledge. Notably, while XAI helps build user trust in the AI systems and provides useful insights for AI developers, trustworthy AI is a different concept. Trustworthiness encompasses not only technologies but also practices, and it has been institutionalized in policy documents guiding AI innovation, such as the EU's High Level Expert Group on AI. To develop a historical view and a perspective on biomedical XAI, we started by reviewing citations from PubMed that fit the above scope definition.

PubMed is a free database of citations of publications in the biomedical domain that was developed by the National Center for Biotechnology Information (NCBI). It is a major source of literature analysis for biomedical researchers due to its comprehensiveness and accessibility. We performed a systematic review of PubMed papers following the recommendations of the PRISMA statement.[34] We applied manual and automatic methods as

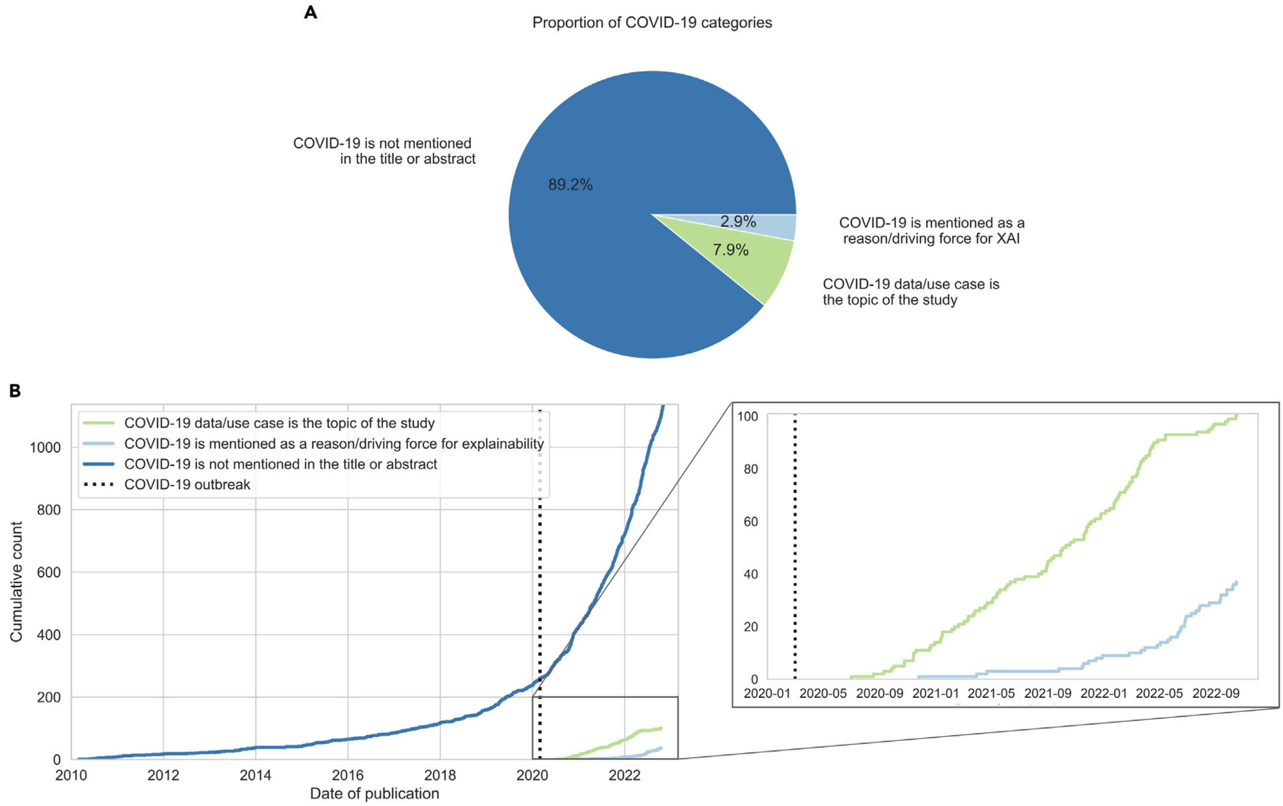


**Figure 1. Trends in XAI papers related to COVID-19**

(A) Proportions of COVID-19 categories in the dataset.

(B) Cumulative number of XAI papers for each category throughout time. The increase in COVID-19-related papers, represented by the light blue and light green curves, set off around 7 months after the pandemic onset.

illustrated in Figure S1; that is, we searched in the PubMed database for all citations until November 3, 2022, with no restrictions on regions, languages, or publication types. Citations were added to our cohort if they contained biomedical concepts in the abstracts or titles, which were automatically extracted based on a prespecified set of keywords (Table S1; Data S1). We excluded studies that were not related to AI and limited the search space with a start date of January 1, 2010 (defined based on the step change of significant attention to the advances of deep-learning technologies). Lastly, we included only papers that were related to XAI, considered as the wide term that includes concepts of the two broad and overlapping subjects discussed above: explainability and causal analysis. The complete information regarding the citation collection criteria used in the data collection process is illustrated in Figure S2. Manual review of the abstracts complemented the automatic extraction. The result is a cohort of 1,276 peer-reviewed papers that study biomedical XAI. In the next section, we discuss research trends that we found in our analysis.

## BIOMEDICAL XAI: A HISTORICAL PERSPECTIVE THROUGH THE LENS OF COVID-19

To investigate the trend of biomedical XAI and the possible role of the COVID-19 pandemic in bringing this field to the forefront of AI research, we used the publication date and constructed a time series defined by the number of biomedical XAI PubMed papers published per month. We focused on the date of COVID-19 outbreak, which was considered March 1, 2020. 261 abstracts on biomedical XAI were published before the outbreak and 1,017 after. After careful selection of studies and iterative manual revision of abstracts, we investigated whether publications related to causality and explainability showed the same trends pre- and post-COVID-19 (Figure S3). There was a much stronger growth in the rate of explainability publications than in causality, which could possibly be due to the difficulties in performing causal inference versus how convenient explaining an existing model is.

We then manually annotated the role of COVID-19 in each paper, where each annotator had three options to label a study: (1) studies that do not mention the pandemic in their titles or abstracts, (2) studies that used COVID-19 data (e.g., chest radiographs or computed tomography [CT] scans, number of SARS-CoV-2 infections, or administrated vaccines) to predict clinical outcomes, or (3) studies that mentioned COVID-19 as a reason or driving force for their research. Expectedly, the distribution of papers per label was uneven (Figure 1A): 1,137 studies (approximately 90%) did not mention the pandemic, whereas 101 studies (8%) used COVID-19 data and 37 studies mentioned COVID-19 as a driving force for their research. Interestingly, COVID-19-related publications significantly increased several months after the pandemic outbreak (Figure 1B, zoomed-in

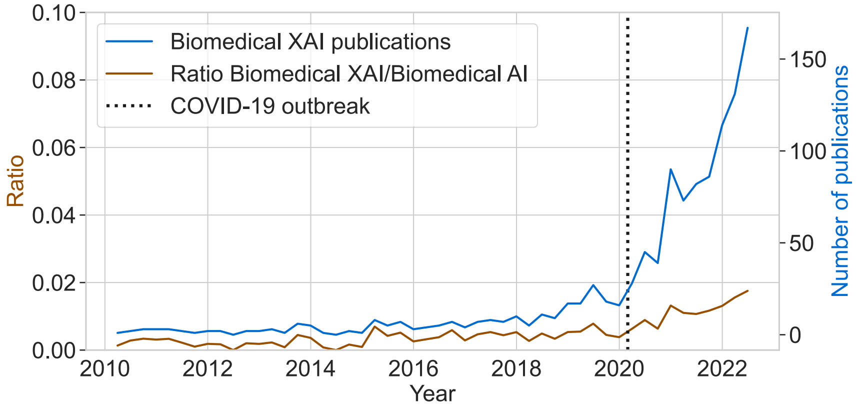


**Figure 2. Comparison between the overall number of biomedical XAI publications (blue curve) and its proportion within the biomedical AI field (brown curve)**
Both plots show quarterly patterns of publications throughout time smoothed by a 1-year rolling average window.

image), possibly representing the time period for researchers to collect enough data, design the studies, and start publishing them.

As an exploratory analysis, we investigated whether a change point is detected at the time of the pandemic outbreak or in the following months (Data S2). We found an inflection period in October 2020, when the quantity of XAI research in biomedical sciences surged upward significantly (Figure S4A). Pinpointing a specific cause for this acceleration is far from trivial. In the past 10 years, XAI has received increased attention in the form of funding, research programs, and legal and ethical requirements. Examples include the DARPA program[35] or the European Commission's White Paper on AI.[36] However, we speculate that the advent of the COVID-19 pandemic itself could have been an important factor in bringing the field of XAI to the forefront in many AI-related research fields. The well-documented difficulties of AI (lack of impact of AI) in COVID-19 analysis could have motivated the need to better understand how models work and the origin of predictions.[12,13] It is worth noting that across the most cited papers we reviewed, often the leading author (last co-author) was an AI researcher that expanded their horizon to the applied biomedical field.[37,38]

We then attempted to quantify the change observed and estimated that the field of biomedical XAI was pushed forward by about 25 months (Figure S4B). However, establishing the causal nature of this link would require a large-scale analysis of multiple research topics trends, which is beyond the scope of the current meta-analysis.

Finally, we compared the numbers of biomedical XAI publications to the ratio between biomedical XAI and biomedical AI publications a long time (Figure 2). We found an exponential growth of biomedical XAI papers, which is followed by an increase in the ratio of biomedical XAI/biomedical AI papers. This is a faster exponential growth than the one detected in publications of AI in biomedical sciences in previous studies,[39] as reflected by the constant increase in the ratio of biomedical XAI/biomedical AI papers. Importantly, the rapid increase in XAI publications is not only due to the increase in scientific literature using COVID-19 data (see, for instance, the exponential growth of the use of medical imaging and AI in the context of COVID-19[40]). On the contrary, our manual review showed that only 8% of the biomedical XAI papers were devoted to analysis of COVID-19 data, whereas 3% (37 papers) referred to COVID-19 as a motivation to discuss or apply XAI methods. This further corroborates our hypothesis that the advent of the COVID-19 pandemic acted as a catalyst and contributed to pushing the level of interest in XAI methodologies to a critical point in an already evolving trend.

The papers list, the manual annotation, and the code used for all analysis can be found in Fghisoni and Visonà.[41]

## BIOMEDICAL XAI: STUDY TYPES

In order to get a more comprehensive view of the type of research performed in biomedical XAI, each abstract was annotated according to the type of study reported in the paper. We followed the approach discussed in Vilone and Longo[25] and labeled the abstracts with one of the following categories that best described the study: (1) review or meta-analysis, (2) discussion of XAI concepts, (3) introduction of novel XAI methods, or (4) evaluation or application of XAI methods and added a fifth category, (5) datasets or tools that support XAI.

In total, 642 papers (approximately 50% of the eligible studies) evaluated or applied existing XAI methods in their respective studies, followed by 214 papers (17%) that introduced a novel XAI technology (either by innovating the way they explain their AI algorithms' decisions or by adapting previous XAI ideas to a new research context; Figure 3A). Letters, comments, and narratives discussing XAI technologies comprised 13% of the cohort. Datasets or tools that support XAI technologies (e.g., software packages, AI frameworks and workflows, medical image datasets) and reviews on XAI methods composed together approximately 20% of the entire cohort.

We then tracked the Google Scholar citations of all eligible publications, considered as a proxy for public interest, and computed the number of citations per month since the date of publication (Figure 3B). As expected, review papers and novel methods attract most attention, as reflected by average citations per month, whereas application and evaluation of methods draw less attention. The relatively high average number of citations of papers with conceptual discussion is somewhat surprising and possibly reflects the heated debate on the role of XAI and its importance in the biomedical domain.

## BIOMEDICAL XAI: FUTURE PROSPECTS

The debate around XAI is an ongoing effort that requires input from experts in computer science, philosophy, ethics, and law. The largest challenges arise from the difficulty of defining and quantifying "explainability": how do we decide if the output of a model is interpretable? A very deep decision tree model, for

CellPress
OPEN ACCESS

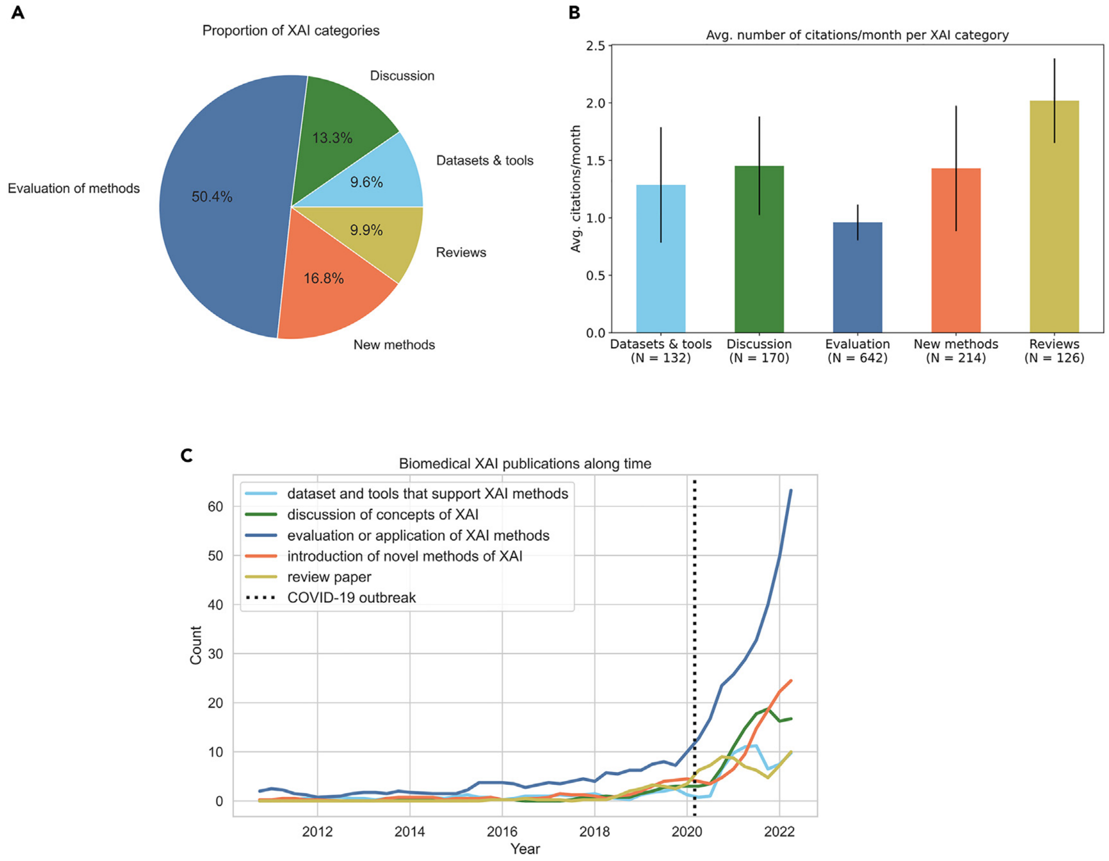


**Figure 3. Biomedical XAI statistics and trends of the cohort of eligible papers**
(A) Proportions of biomedical XAI categories.
(B) Average number of citations per month since published date. Error bars represent 95% confidence intervals.
(C) Counts of biomedical XAI papers published per quarter year. For each category (colored curve), we smoothed quarterly patterns by using the 1 year rolling average window.

example, would offer a direct explanation for the predicted outcome while being useless for human interpretation due to the sheer amount of split choices presented. And yet, despite these obstacles, ongoing discussion is necessary for the advancement of AI, especially in high-stakes settings, not only to improve robustness and reliability but also to satisfy the growing levels of legal requirements for the "right of explanation" that is being adopted to protect the stakeholders involved.[42]

Briefly, XAI algorithms can be divided into two main categories: transparent models that convey some intrinsic degree of interpretability and post hoc explainability techniques. One of the most prominent directions in the field is progressing toward leveraging the latter techniques, where explanation is provided to existing opaque models rather than the *de novo* design of methods rooted in causality. When analyzing the content of the ten most popular papers (based on their average citations), it was observed that Lundberg et al.'s study[43] was considerably the most cited one, averaging over 60 citations per month since its publication. Among these papers, four of them,[43–46] including Lundberg and colleagues' work, present post hoc explainability methods, while the remaining six papers[47–52] focus on developing inherently interpretable models. This tendency to explore post hoc solution could stem from the fact that several explanation techniques can be applied to a variety of models with minimal added efforts. Lundberg's Shapley additive explanations (SHAP) analysis was frequently used in the biomedical literature that we reviewed. Although the majority of current post hoc methods only provide locally reliable explanations and can be misleading of model functionality,[53] Shapley values could be the sole approach that complies with legal requirements due to its foundation in a solid theory derived from axioms of a fair game.[54]

Above these variety of approaches, there is an ongoing debate regarding the tradeoff between performance and interpretability of machine-learning models. Widespread in the literature is the opinion that retaining or designing interpretability into an ML solution comes at a cost in accuracy of the predictions. It has been argued that such a tradeoff is but a myth,[55] and to an extent this holds true. Often, different models, including explainable ones, offer very similar performances.[56] Within these settings, it is therefore obvious to choose the solution that is also easier to interpret.

However, recent advances in large-scale deep-learning models have challenged this idea once again. The successes of foundation models,[57] which are those models trained on broad data and then adapted to specific tasks, have put in

question whether any explainable model could reach the same level of performance. The results produced by BERT,[58] GPT-3,[59] and DALL-E[60] are outclassing those of any transparent model by a wide margin. Recently, it was shown that large language models such as ChatGPT[61] may have the potential to assist with clinical reasoning.[62] Hence, it might become a tool to explain AI-based approaches and support AI-based decisions.

The paradigm shift produced by foundation models can offer completely different opportunities to make use of interpretations of their results. While a common approach with simpler models would be to diagnose the elements that lead to specific predictions (e.g., analyze the importance of features), with large-scale models, it is possible to obtain novel designs derived from the surprising emergent properties learned from the sheer quantity of data, which could expand our understanding of the task at hand in completely unexpected directions. Within the biomedical field, this could lead to, for example, the design of novel proteins that are not observed in nature[63] or to exploring new connections between diseases.[64]

Are the post hoc explainable technologies satisfying? Does the success of large-scale deep-learning models mean that it is futile to pursue non-post hoc explainability in AI? Perhaps it represents an unreachable goal. However, the increase in BXAI publications, highlighted in our survey, indicates that many research groups believe that the future of AI-based advances in medicine and health belongs to those who successfully address such questions.

## DATA AND CODE AVAILABILITY

All data analyzed and produced in this study, as well as the original code, have been deposited at https://github.com/gvisona/BiomedXAI and at the repository in Zenodo (https://doi.org/10.5281/zenodo.8207487). Data are publicly available as of the date of publication.

### SUPPLEMENTAL INFORMATION

Supplemental information can be found online at https://doi.org/10.1016/j.patter.2023.100830.

### ACKNOWLEDGMENTS

We are extremely grateful for Prof. Chris Holmes for his critical reading and valuable comments. We acknowledge the funding received from the European Union's Framework Programme for Research and Innovation Horizon 2020 (2014–2020) under the Marie Skłodowska-Curie Grant agreement no. 813533-MSCA-ITN-2018. I.S. was funded by the "DDAI" COMET Module within the COMET – Competence Centers for Excellent Technologies Programme, funded by the Austrian Federal Ministry for Transport, Innovation and Technology (BMVIT), the Austrian Federal Ministry for Digital and Economic Affairs (BMDW), the Austrian Research Promotion Agency (FFG), the Province of Styria (SFG), and partners from industry and academia. The COMET Program is managed by FFG. Finally, we acknowledge the Big Data Value Association (BDVA), Brussels, Belgium.

### AUTHOR CONTRIBUTIONS

Conceptualization of the study was conducted by M.R.-Z. Methodology was led by G.S., G.V., L.M., M.L., M.R.-Z., and V.B. and was conducted by all authors. Data curation was done by B.E.V., C.F., C.J.-Q., F.G., F.M., G.V., I.S., L.G., L.M., M.L., M.R.-Z., P.J.N., R.K., S.S., and V.B. Formal analysis was conducted by G.V., M.R.-Z., and V.B. Investigation was equally conducted by all authors. Project administration was done by M.L. and V.B. Resources and software was done by B.E.V., F.G., G.V., L.M., and V.B. Supervision was done by M.R.-Z. Validation, visualization, and writing of original draft were done by G.S., G.V., M.R.-Z., L.G., L.M., P.J.N., R.K., and V.B. Writing (review & editing) was equally done by all authors.

### DECLARATION OF INTERESTS

M.R.-Z. and V.B. are employees of IBM Research, Haifa, Israel. F.M. is an employee of Philips Research, Eindhoven, the Netherlands. I.S. has received funding from multiple funding agencies through a collaborative funding program and declares no support from any organization for the submitted work. P.J.N. receives funding from the Dutch Research Council (DWO) for the grant "Mobile Support Systems for Behavior Change," of which he is the principal investigator (P.I.). M.L. is funded by the EU-Commission grant no. 101057497-EDIAQI.

### REFERENCES

1. Tanne, J.H. (2021). Covid-19: FDA approves Pfizer-BioNTech vaccine in record time. BMJ *374*, n2096.

2. (2022). A critical overview of current progress for COVID-19: development of vaccines, antiviral drugs, and therapeutic antibodies. J. Biomed. Sci. *29*, 68. https://doi.org/10.1186/s12929-022-00852-9. https://link.springer.com/article/10.1186/s12929-022-00852-9.

3. Addressing Backlogs and Managing Waiting Lists during and beyond the COVID-19 Pandemic [Internet]. [cited 2023 Jan 11]. Available from: https://eurohealthobservatory.who.int/publications/i/addressing-backlogs-and-managing-waiting-lists-during-and-beyond-the-covid-19-pandemic.

4. Haldane, V., De Foo, C., Abdalla, S.M., Jung, A.S., Tan, M., Wu, S., Chua, A., Verma, M., Shrestha, P., Singh, S., et al. (2021 Jun). Health systems resilience in managing the COVID-19 pandemic: lessons from 28 countries. Nat. Med. *27*, 964–980.

5. Martin, A., Nateqi, J., Gruarin, S., Munsch, N., Abdarahmane, I., Zobel, M., and Knapp, B. (2020). An artificial intelligence-based first-line defence against COVID-19: digitally screening citizens for risks via a chatbot. Sci. Rep. *10*, 19012.

6. Wang, M., Xia, C., Huang, L., Xu, S., Qin, C., Liu, J., Cao, Y., Yu, P., Zhu, T., Zhu, H., et al. (2020). Deep learning-based triage and analysis of lesion burden for COVID-19: a retrospective study with external validation. Lancet Digit. Health *2*, e506–e515.

7. Jayatunga, M.K.P., Xie, W., Ruder, L., Schulze, U., and Meier, C. (2022). AI in small-molecule drug discovery: a coming wave? Nat. Rev. Drug Discov. *21*, 175–176.

8. Palmisano, A., Vignale, D., Boccia, E., Nonis, A., Gnasso, C., Leone, R., Montagna, M., Nicoletti, V., Bianchi, A.G., Brusamolino, S., et al. (2022). AI-SCoRE (artificial intelligence-SARS CoV2 risk evaluation): a fast, objective and fully automated platform to predict the outcome in COVID-19 patients. Radiol. Med. *127*, 960–972.

9. Ekman, I., Busse, R., van Ginneken, E., Van Hoof, C., van Ittersum, L., Klink, A., Kremer, J.A., Miraldo, M., Olauson, A., De Raedt, W., et al. (2016). Health-care improvements in a financially constrained environment. Lancet *387*, 646–647.

10. Shen, J., Zhang, C.J.P., Jiang, B., Chen, J., Song, J., Liu, Z., He, Z., Wong, S.Y., Fang, P.H., and Ming, W.K. (2019). Artificial Intelligence Versus Clinicians in Disease Diagnosis: Systematic Review. JMIR Med. Inform. *7*, e10010.

11. Rajpurkar, P., Chen, E., Banerjee, O., and Topol, E.J. (2022). AI in health and medicine. Nat. Med. *28*, 31–38.

12. Roberts, M., Driggs, D., Thorpe, M., Gilbey, J., Yeung, M., Ursprung, S., Aviles-Rivero, A.I., Etmann, C., McCague, C., Beer, L., et al. (2021). Common pitfalls and recommendations for using machine learning to detect and prognosticate for COVID-19 using chest radiographs and CT scans. Nat. Mach. Intell. *3*, 199–217.

13. Wynants, L., Van Calster, B., Collins, G.S., Riley, R.D., Heinze, G., Schuit, E., Albu, E., Arshi, B., Bellou, V., Bonten, M.M.J., et al. (2020). Prediction

CellPress
OPEN ACCESS

models for diagnosis and prognosis of covid-19: systematic review and critical appraisal. BMJ *369*, m1328.

14. Kaufman, S., Rosset, S., Perlich, C., and Stitelman, O. (2012). Leakage in data mining: Formulation, detection, and avoidance. ACM Trans. Knowl. Discov. Data *6*, 1–21.

15. Salahuddin, Z., Woodruff, H.C., Chatterjee, A., and Lambin, P. (2022). Transparency of deep neural networks for medical image analysis: A review of interpretability methods. Comput. Biol. Med. *140*, 105111.

16. Miller, T. (2019). Explanation in artificial intelligence: Insights from the social sciences. Artif. Intell. *267*, 1–38.

17. Adadi, A., and Berrada, M. (2018). Peeking Inside the Black-Box: A Survey on Explainable Artificial Intelligence (XAI). IEEE Access *6*, 52138–52160.

18. Doran, D., Schulz, S., and Besold, T.R. (2017). What Does Explainable AI Really Mean? A New Conceptualization of Perspectives. Preprint at arXiv. https://doi.org/10.48550/arXiv.1710.00794.

19. Markus, A.F., Kors, J.A., and Rijnbeek, P.R. (2021). The role of explainability in creating trustworthy artificial intelligence for health care: A comprehensive survey of the terminology, design choices, and evaluation strategies. J. Biomed. Inf. *113*, 103655.

20. Cutillo, C.M., Sharma, K.R., Foschini, L., Kundu, S., Mackintosh, M., Mandl, K.D., Beck, T., Collier, E., Colvis, C., Gersing, K., et al. (2020). Machine intelligence in healthcare—perspectives on trustworthiness, explainability, usability, and transparency. npj Digit. Med. *3*, 47–55.

21. (2020). Explainability for artificial intelligence in healthcare: a multidisciplinary perspective. BMC Med. Inf. Decis. Making *20*, 310. https://doi.org/10.1186/s12911-020-01332-6. https://bmcmedinformdecismak.biomedcentral.com/articles/10.1186/s12911-020-01332-6.

22. Payrovnaziri, S.N., Chen, Z., Rengifo-Moreno, P., Miller, T., Bian, J., Chen, J.H., Liu, X., and He, Z. (2020). Explainable artificial intelligence models using real-world electronic health record data: a systematic scoping review. J. Am. Med. Inf. Assoc. *27*, 1173–1185.

23. Barredo Arrieta, A., Díaz-Rodríguez, N., Del Ser, J., Bennetot, A., Tabik, S., Barbado, A., Garcia, S., Gil-Lopez, S., Molina, D., Benjamins, R., et al. (2020). Explainable Artificial Intelligence (XAI): Concepts, taxonomies, opportunities and challenges toward responsible AI. Inf. Fusion *58*, 82–115.

24. Das, A., and Rad, P. (2020). Opportunities and Challenges in Explainable Artificial Intelligence (XAI): A Survey. Preprint at arXiv. https://doi.org/10.48550/arXiv.2006.11371.

25. Vilone, G., and Longo, L. (2020). Explainable Artificial Intelligence: a Systematic Review. Preprint at arXiv. https://doi.org/10.48550/arXiv.2006.00093.

26. Jiménez-Luna, J., Grisoni, F., and Schneider, G. (2020). Drug discovery with explainable artificial intelligence. Nat. Mach. Intell. *2*, 573–584.

27. Tjoa, E., and Guan, C. (2021). A Survey on Explainable Artificial Intelligence (XAI): Toward Medical XAI. IEEE Transact. Neural Networks Learn. Syst. *32*, 4793–4813.

28. Loh, H.W., Ooi, C.P., Seoni, S., Barua, P.D., Molinari, F., and Acharya, U.R. (2022). Application of explainable artificial intelligence for healthcare: A systematic review of the last decade (2011–2022). Comput. Methods Progr. Biomed. *226*, 107161.

29. Byrne, R.M.J. (2019). Counterfactuals in Explainable Artificial Intelligence (XAI): Evidence from Human Reasoning. In Proceedings of the Twenty-Eighth International Joint Conference on Artificial Intelligence (International Joint Conferences on Artificial Intelligence Organization) https://www.ijcai.org/proceedings/2019/876.

30. Kusner, M.J., Loftus, J., Russell, C., and Silva, R. (2017). Counterfactual Fairness. In Advances in Neural Information Processing Systems (Curran Associates, Inc.) https://proceedings.neurips.cc/paper/2017/hash/a486cd07e4ac3d270571622f4f316ec5-Abstract.html.

31. (2019). Causability and explainability of artificial intelligence in medicine. Wiley Interdiscip Rev Data Min Knowl Discov. WIREs Data Mining and Knowledge Discovery. https://wires.onlinelibrary.wiley.com/doi/full/10.1002/widm.1312.

32. (2022). Causal machine learning for healthcare and precision medicine. Preprint at arXiv. Royal Society Open Science. https://doi.org/10.48550/arXiv.2205.11402.

33. Rajkomar, A., Dean, J., and Kohane, I. (2019). Machine Learning in Medicine. N. Engl. J. Med. *380*, 1347–1358.

34. Moher, D., Liberati, A., Tetzlaff, J., and Altman, D.G. (2009). Preferred reporting items for systematic reviews and meta-analyses: the PRISMA statement. BMJ *6*, e1000097.

35. Gunning, D., and Aha, D.W. (2019). DARPA's Explainable Artificial Intelligence (XAI) Program. AI Mag. *40*, 44–58.

36. Paper on Artificial Intelligence: A European Approach to Excellence and Trust [Internet]. [cited 2023 Jun 13]. Available from: https://commission.europa.eu/publications/white-paper-artificial-intelligence-european-approach-excellence-and-trust_en.

37. Wu, Y.H., Gao, S.H., Mei, J., Xu, J., Fan, D.P., Zhang, R.G., and Cheng, M.M. (2021). JCS: An Explainable COVID-19 Diagnosis System by Joint Classification and Segmentation. IEEE Trans. Image Process. *30*, 3113–3126.

38. Linardatos, P., Papastefanopoulos, V., and Kotsiantis, S. (2020). Explainable AI: A Review of Machine Learning Interpretability Methods. Entropy *23*, 18.

39. Tran, B., Vu, G., Ha, G., Vuong, Q.H., Ho, M.T., Vuong, T.T., La, V.P., Ho, M.T., Nghiem, K.C., Nguyen, H., et al. (2019). Global Evolution of Research in Artificial Intelligence in Health and Medicine: A Bibliometric Study. J. Clin. Med. *8*, 360.

40. Born, J., Beymer, D., Rajan, D., Coy, A., Mukherjee, V.V., Manica, M., Prasanna, P., Ballah, D., Guindy, M., Shaham, D., et al. (2021). On the role of artificial intelligence in medical imaging of COVID-19. Patterns (N. Y). *2*, 100330.

41. Fghisoni, and Visonà, G. (2023). Gvisona/BiomedXAI: BiomedXAI - Patterns Submission v1.0 (v1.0) (Zenodo). https://doi.org/10.5281/ZENODO.8207487.

42. Aranovich, T.d.C., and Matulionyte, R. (2022). Ensuring AI explainability in healthcare: problems and possible policy solutions. Inf. Commun. Technol. Law *32*, 259–275.

43. Lundberg, S.M., Erion, G., Chen, H., DeGrave, A., Prutkin, J.M., Nair, B., Katz, R., Himmelfarb, J., Bansal, N., and Lee, S.I. (2020). From local explanations to global understanding with explainable AI for trees. Nat. Mach. Intell. *2*, 56–67.

44. Lee, H., Yune, S., Mansouri, M., Kim, M., Tajmir, S.H., Guerrier, C.E., Ebert, S.A., Pomerantz, S.R., Romero, J.M., Kamalian, S., et al. (2018). An explainable deep-learning algorithm for the detection of acute intracranial haemorrhage from small datasets. Nat. Biomed. Eng. *3*, 173–182.

45. Lauritsen, S.M., Kristensen, M., Olsen, M.V., Larsen, M.S., Lauritsen, K.M., Jørgensen, M.J., Lange, J., and Thiesson, B. (2020). Explainable artificial intelligence model to predict acute critical illness from electronic health records. Nat. Commun. *11*, 3852.

46. Nagasubramanian, K., Jones, S., Singh, A.K., Sarkar, S., Singh, A., and Ganapathysubramanian, B. (2019). Plant disease identification using explainable 3D deep learning on hyperspectral images. Plant Methods *15*, 98.

47. Yang, J.H., Wright, S.N., Hamblin, M., McCloskey, D., Alcantar, M.A., Schrübbers, L., Lopatkin, A.J., Satish, S., Nili, A., Palsson, B.O., et al. (2019). A White-Box Machine Learning Approach for Revealing Antibiotic Mechanisms of Action. Cell *177*, 1649–1661.e9.

48. Carter, H., Douville, C., Stenson, P.D., Cooper, D.N., and Karchin, R. (2013). Identifying Mendelian disease genes with the Variant Effect Scoring Tool. BMC Genom. *14*, S3.

49. Tank, A., Covert, I., Foti, N., Shojaie, A., and Fox, E.B. (2022). Neural Granger Causality. IEEE Trans. Pattern Anal. Mach. Intell. *44*, 4267–4279.

50. Zhou, X., Zhou, J., Xin, F., Ma, J., Zhang, W., Wu, H., Jiang, M., and Dong, W. (2018). Deep learning sequence-based ab initio prediction of variant effects on expression and disease risk. Nat. Genet. *119*, 1171–1178.

51. Han, Y., Chen, C., Tewfik, A., Ding, Y., and Peng, Y. (2021). Pneumonia Detection On Chest X-Ray Using Radiomic Features And Contrastive Learning. In 2021 IEEE 18th International Symposium on Biomedical Imaging (ISBI)), pp. 247–251.

52. Smedley, D., Schubach, M., Jacobsen, J., Köhler, S., Zemojtel, T., Spielmann, M., Jäger, M., Hochheiser, H., Washington, N., McMurry, J., et al. (2016). A Whole-Genome Analysis Framework for Effective Identification of Pathogenic Regulatory Variants in Mendelian Disease. Am. J. Hum. Genet. *99*, 595–606.

53. Mittelstadt, B., Russell, C., and Wachter, S. (2019). Explaining Explanations in AI. In Proceedings of the Conference on Fairness, Accountability, and Transparency, pp. 279–288. http://arxiv.org/abs/1811.01439.

54. Molnar, C. (2023), Interpretable Machine Learning. https://christophm.github.io/interpretable-ml-book/.

55. Rudin, C. (2019). Stop explaining black box machine learning models for high stakes decisions and use interpretable models instead. Nat. Mach. Intell. *1*, 206–215.

56. Fernández-Delgado, M., Cernadas, E., Barro, S., and Amorim, D. (2014). Do we Need Hundreds of Classifiers to Solve Real World Classification Problems? J. Mach. Learn. Res. *15*, 3133–3181.

57. Bommasani, R., Hudson, D.A., Adeli, E., Altman, R., Arora, S., von Arx, S., et al. (2022). On the Opportunities and Risks of Foundation Models. Preprint at arXiv. https://doi.org/10.48550/arXiv.2108.07258. http://arxiv.org/abs/2108.07258.

58. Devlin, J., Chang, M.W., Lee, K., and Toutanova, K. (2019). BERT: Pre-training of Deep Bidirectional Transformers for Language Understanding. Preprint at arXiv. https://doi.org/10.48550/arXiv.1810.04805. http://arxiv.org/abs/1810.04805.

59. Brown, T.B., Mann, B., Ryder, N., Subbiah, M., Kaplan, J., Dhariwal, P., et al. (2020). Language Models are Few-Shot Learners. Preprint at arXiv. https://doi.org/10.48550/arXiv.2005.14165. http://arxiv.org/abs/2005.14165.

60. Ramesh, A., Pavlov, M., Goh, G., Gray, S., Voss, C., Radford, A., et al. (2021). Zero-Shot Text-to-Image Generation. Preprint at arXiv. https://doi.org/10.48550/arXiv.2102.12092. http://arxiv.org/abs/2102.12092.

61. ChatGPT. https://openai.com/blog/chatgpt.

62. Kung, T.H., Cheatham, M., ChatGPT, Medenilla, A., Sillos, C., Leon, L.D., et al. (2022). Performance of ChatGPT on USMLE: Potential for AI-Assisted Medical Education Using Large Language Models. Preprint at medRxiv. https://doi.org/10.1101/2022.12.19.22283643. https://www.medrxiv.org/content/10.1101/2022.12.19.22283643v2.

63. Ferruz, N., Schmidt, S., and Höcker, B. (2022). ProtGPT2 is a deep unsupervised language model for protein design. Nat. Commun. *13*, 4348.

64. Li, Y., Rao, S., Solares, J.R.A., Hassaine, A., Ramakrishnan, R., Canoy, D., Zhu, Y., Rahimi, K., and Salimi-Khorshidi, G. (2020). BEHRT: Transformer for Electronic Health Records. Sci. Rep. *10*, 7155.

## About the author

**Luca Maliverno** holds a PhD in physics. He is a data scientist in Porini SRL and a Microsoft Certified Trainer. He is part of the Porini BU Education, where he takes care of the Porini Innovation and Research Center for DataScience (PIRC). His interests lie in artificial intelligence for business application and digital transformation.

**Vesna Barros** is a research scientist at IBM Research. She graduated in electrical engineering at the University of Brasilia (BSc) and ETH Zurich (MSc). Before joining IBM, she worked as a data scientist at Novartis and Philips Healthcare. She carries out research at the intersection of machine learning and medicine with the goal of improving diagnosis and treatment outcomes to the benefit of the care and wellbeing of patients. She is currently pursuing a PhD at the Hebrew University of Jerusalem, Israel.

**Francesco Ghisoni** is a PhD candidate in quantum machine learning at the University of Pavia. He holds a master's degree in physics from King's College London, where his thesis focused on quantum Monte Carlo methods. Francesco has extensive international experience, having completed a 1 year fellowship at the University of California Berkeley and a research period in Canada at the Institute for Quantum Computing. He has won numerous awards, including recognition for academic achievement at KCL and first place at the ETH Quantum Hackathon in 2023. His professional experience includes working at Porini as a data science trainer and researcher.

**Giovanni Visonà** graduated in physics at the Universities of Turin (BSc) and Trent (MSc). After obtaining his MSc in artificial intelligence at the University of Edinburgh, he joined in December 2019 the Marie Curie Innovative Training Network "Machine Learning Frontiers in Precision Medicine." He is currently pursuing a PhD at the Max-Planck Institute for Intelligent Systems in Tübingen, Germany.

**Roman Kern** is chief scientific officer at Know-Center, a research center for trustworthy AI, and works at the Institute for Interactive Systems and Data Science at the Technical University of Graz. He was awarded his PhD in computer science by the Graz University of Technology. His research interests include natural language processing and machine learning, with a focus on causal data science. He applies these approaches in various application fields with the goal to achieve trustworthy AI methods.

**Philip J. Nickel** (PhD UCLA, 2002) is an associate professor in the philosophy and e thics group at Eindhoven University of Technology. He specializes in the philosophy of trust and the ethics of disruptive technologies. Some of his research is in the domain of biomedical ethics, focusing on issues of consent and the mediation of care through technology and data. He leads the project "Mobile Support Systems for Behavior Change" funded by the Dutch Research Council's Responsible Innovation program.

**Barbara Elvira Ventura** is a data analyst at Porini SRL. She holds a degree in bioengineering and biomedical engineering from University of Pavia.

**Ilija Šimić** is a researcher at Know-Center GmbH, Austria. He earned his bachelor's and master's degree in computer science at Graz University of Technology. In his research, he investigates explainable AI methods for debugging, verifying, and extracting knowledge from state-of-the-art deep-learning models for time series classification.

**Sarah Stryeck** has a PhD in biophysics and metabolism research from the Medical University of Graz, Austria. After her PhD, she worked for 3 years in data management/data governance at the Graz University of Technology and at Know-Center GmbH. Currently, Sarah is head of digitalization and data governance at the Research Center Pharmaceutical Engineering GmbH in Graz, Austria, and is consulting the Data Intelligence Initiative as data steward expert.

**Francesca Manni** is currently a clinical scientist at Philips. She has a background in biomedical engineering with a focus on AI for medical imaging. During her PhD at TUe Eindhoven, she focused on the development of novel imaging technologies for tumor detection and minimally invasive surgery. After that, she was an AI and data scientist at Philips Research. She has worked on enabling AI solutions in healthcare while leading the healthcare group at the BDVA. Francesca's research is reported in numerous international peer-reviewed scientific journals and top international conference proceedings in the field of image-guided interventions and AI.

**Cèsar Ferri** is a professor in the Department of Computer Systems and Computation at the èèUniversitat Politènica de València. He is also a member of the Valencian Graduate School and Research Network of Artificial Intelligence (ValgAI). Furthermore, he holds a long-standing research position in the DMIP team (a subgroup of VRAIN), which commenced in 1999. His research interest orients around machine learning and artificial intelligence. Cèsar has published papers in leading journals and at top conferences as a co-author on the aforementioned topics.

**Claire Jean-Quartier** has a PhD in the field of preclinical research regarding cell signaling with a master's in environmental system sciences and chemistry, as well as in molecular biomedical sciences. C.J. has been working in chemical, pharmaceutical, and environmental analytics and quality assurance and is engaged in bioinformatical cancer research and explainable AI at the Human-Centered AI Lab. As well as focusing on sustainability aspects in interdisciplinary research, she is currently working at the Graz University of Technology, fostering open science and research data management.

**Laura Genga** is an assistant professor in information systems at the Eindhoven University of Technology (TU/e). Her research revolves around

data-driven process analysis and enhancement. In particular, her work falls within the process mining discipline, the goal of which consists of turning event data collected by any sort of logging system, e.g., enterprise information systems, into insights on the corresponding processes. Her core topics involve automated discovery and analysis of flexible processes, process compliance analysis, and online and monitoring and prediction to support process managers in making decisions regarding current process executions, e.g., to react to potentially undesired situations.

**Dr. Gabriele Schweikert** previously completed a PhD at the Max Planck Institute Tuebingen (Schoelkopf, Weigel, Raetsch labs), developing machine-learning techniques for computational gene finding. She then moved on to the Wellcome Trust Center for Cell Biology in Edinburgh, joining the lab of Adrian Bird, one of the pioneers of epigenomic research. She later held a Marie Curie and an EMBO Fellowship at the School of Informatics, University of Edinburgh, prior to coming to the Division of Computational Biology at the School of Life Sciences, University of Dundee.

**Dr.sc. Mario Lovrić** leads the laboratory for chemical and biomedical informatics at the Institute for Anthropological Research in Zagreb and teaches as an assistant professor at the Faculty of Electrical Engineering in Osijek, both in Croatia. He is also scientific director of the Horizon EDIAQI project funded by the European Commission. Mario holds a PhD in computational chemistry with a focus on toxicology and has conducted his post-doc research in Know-Center, Austria, and RegionH, Denmark. Mario has published more than 30 peer-reviewed papers.

**Prof. Michal Rosen-Zvi** is the director of AI for drug discovery at IBM Research and an adjunct computational medicine professor at the Hebrew University at the Faculty of Medicine. She is also heading the AI for Accelerated HC&LS Discovery department at IBM Research, Israel. Michal holds a PhD in computational physics and completed her postdoctoral studies at UC Berkeley in machine learning. She joined IBM Research in 2005. Michal has published more than 50 peer-reviewed papers. She has served as a reviewer and guest editor in various leading conferences and journals and has served on various boards.

Patterns, Volume *4*

## Supplemental information

# A historical perspective

# of biomedical explainable AI research

**Luca Malinverno, Vesna Barros, Francesco Ghisoni, Giovanni Visonà, Roman Kern, Philip J. Nickel, Barbara Elvira Ventura, Ilija Šimić, Sarah Stryeck, Francesca Manni, Cesar Ferri, Claire Jean-Quartier, Laura Genga, Gabriele Schweikert, Mario Lovrić, and Michal Rosen-Zvi**

# Data S1

## Section 1: Search strategy

The search strategy in the PubMed database was performed on November 3rd, 2022, and the study flow is illustrated in Figure E1. First, we focused on the automatic extraction of biomedical papers. We consulted with the Physician Specialty Data Report of the Association of American Medical Colleges and selected as keywords of biomedical papers all specialities that have more than 10,000 active physicians (as of 2019). Second, we extracted papers related to XAI within the biomedical sciences. To do so, we focused on concepts of explainability and causal inference. Table E1 provides a summary of the different terminologies we used to access citations of these two groups. The search strategy had the following steps:

1. Explore the full space of all PubMed citations.
2. Extract all citations whose title or abstract refers to biomedical concepts (using the keywords "anesthesiology", "biomedical", "biomedicine", "bioinformatics", "cardiovascular", "clinical", "critical care", "dermatology", "disease", "drug","gastroenterology", "genetics", "genomics", "gynecology", "health", "healthcare", "hematology", "immunology", "life sciences" "medical", "medicine", "nephrology", "neurology", "obstetrics", "oncology","ophthalmology", "orthopedic","pathology", "pediatrics", "pharmacology", "protein", "psychiatry", "radiology", "surgery", "urology", "vaccine").
3. From the subset of biomedical cohort, extract all citations whose title or abstract refer to AI concepts (using the keywords "AI", "artificial intelligence", "deep learning" and "machine learning").
4. Limit the cohort to be composed of papers published since Jan 1, 2010.
5. From the subset above, extract papers related to explainability and/or causal (using the keywords listed in Table 1) while recording which of the three concepts (or combination of them) are used to extract the paper: explainability, causal or both.

Table S1: Keywords used to extract XAI-related abstracts and define the two cohorts: explainability and causal.

| Terminology | Keywords | Explanation |
|---|---|---|
| Explainability | "black box models", "explainable", "interpretability", "explainability", "opacity", "opaque", “xai” | Interpretable and comprehensible models and truly explainable models offering an automated reasoning that explains the decision making process of a model using human-understandable features of the input data [1]. |
| Causal | "causal", "causation", "counterfactual” | Finding out how much would the effect variable(s) change as a result of modifying the value of input variable(s) and discovering which variables could be modified in order to change the value of the effect variable(s)[2]. |

The inclusion/exclusion criteria resulted in the creation of the cohort exemplified in Figure E2. Among all PubMed citations retrieved on November 3rd, 2022 (over 34 million), we excluded the ones not related to biomedical concepts (n = 12·4 million) or AI (n = 21·4 million). Of 148,700 remaining papers, we excluded 27,851 that were published before 2010. This left 120,849 papers, from which 980 referred to explainability, 623 referred to causality and 14 papers that referred to both. We manually reviewed 1,603 abstracts belonging to these two groups with respect to language, category of the paper and the role of COVID-19 in the context of the studies, 1,276 of which met criteria for final inclusion.

When analysing the differences in trends between publications with "causal" terms versus publications with "explainability" terms, we found that they followed different growth trends, as shown in Figure E3.

# Data S2

## Section 1: Trend analysis

We assumed that the number of monthly biomedical XAI publications in general follows an exponential curve, a common assumption when modelling the growing number of publications in a field [3,4]. More specifically, we followed the ideas presented in [5] and modeled changing trends as a piecewise Negative Binomial Regression task. As the data available is counts over time, we deemed a Poisson-like regression method to be more appropriate than a simple exponential fit; due to detected overdispersion, we selected an NB1 Negative Binomial[6] rather than a simple Poisson model. The assumed functional form is:

$$\begin{gathered} y(x) \sim NB(\mu(x), \alpha) \\ \mu(x) = \beta_0 + \beta_1 * x + \beta_2 * \theta_D \\ Var[y|x] = \mu(x) + \alpha\mu(x) \end{gathered} \tag{1}$$

where $y$ is the number of monthly biomedical XAI papers, $x$ is the number of months elapsed since the start of the time series, $a$ is a parameter to be fit and $\theta_D$ is a Heaviside step function that has value 0 for dates before the transition date D, and value one afterwards.

We define the task of detecting a change in trend as the determination of the date $D$ where the threshold indicator $\theta_D$ should change value to best explain the observed data. To find this date, we examine the goodness of fit of Equation 1 for varying change points $D$. Afterwards, we compare the performance of this piecewise exponential fit to a single fitting function (equivalent to setting the parameters $b$ and $d$) using an F-test.

After determining the changepoint $D$, we attempt to interpret it and quantify the observed effect by fitting a different Negative Binomial model, of the form:

$$y(x) \sim NB(\mu(x), \alpha)$$
$$\mu(x) = \beta_0 + \beta_1 * (x + \Delta * \theta_D) \quad (2)$$
$$Var[y|x] = \mu(x) + \alpha\mu(x)$$

Under the assumption that without external intervention the progression of a series should be smooth, this second fitting task is equivalent to asking: "When would we have likely observed the data points after D if the intervention never happened?". The trend changepoint analysis was performed with Python's *scipy* library (version 1·9·3; https://scipy.org) [7] and *statmodels* library (version 0.13.5; https://statsmodels.org) [8].

We fit the Negative Binominal Regression presented in Equation 1 for varying threshold months *D* and evaluated the goodness of the fit using the Coefficient of Determination $R^2$ and the Sum of Squared Errors (SSE, also called Residual Sum of Squares). The results, presented in the upper part of Figure E4, show that the optimal fit is achieved when modelling a change in trend starting from October 2020. The corresponding piecewise fit is shown in the middle portion of the figure.

We compared the piecewise function with a trend change in October 2020 against the single fitting function (with $\theta_D = 0$, or equivalently $\beta_2 = 0$) using an F-test. The resulting F-statistic of 40·9 results in a p-value of $1 * 10^{-63}$, which supports the claim that the improvement brought by the piecewise fit is statistically significant for a threshold of 0·01 even after adjustments for hypothesis testing.

Robustly defining the cause of this trend change is far from trivial, however we hypothesize that the outbreak of the COVID-19 pandemic is the main external contributor to affect an already growing trend. Considering the months necessary to gather and analyze data, and compile the results in a published paper, it is plausible to consider that the effect of the pandemic on the rate of publications would require several months to appear.

We then fixed the detected trend changepoint in October 2020, and fit the regression model in Equation 2, where the effect of the changepoint is modelled as a shift in time. The resulting fit, shown in Figure E4.B, is that the trend change is equivalent to a shift of 25 months of the points observed after October 2020. Under the assumption of smoothness in the absence of interventions, we would have therefore expected to see the rates of publications observed after October 2020 only after August 2022. Equivalently, we can describe the effect of the trend change as pushing forward the rate of publications in biomedical XAI by 25 months.

Supplemental references


1. Doran D, Schulz S, Besold TR. What Does Explainable AI Really Mean? A New Conceptualization of Perspectives [Internet]. arXiv; 2017 [cited 2022 Nov 4]. Available from: http://arxiv.org/abs/1710.00794

2. Guo R, Cheng L, Li J, Hahn PR, Liu H. A Survey of Learning Causality with Data: Problems and Methods. ACM Comput Surv. 2021 Jul 31;53(4):1–37.

3. Parolo PDB, Pan RK, Ghosh R, Huberman BA, Kaski K, Fortunato S. Attention decay in science. Journal of Informetrics. 2015 Oct 1;9(4):734–45.

4. Bornmann L, Mutz R. Growth rates of modern science: A bibliometric analysis based on the number of publications and cited references. Journal of the Association for Information Science and Technology. 2015;66(11):2215–22.

5. Bornmann L, Haunschild R, Mutz R. Growth rates of modern science: a latent piecewise growth curve approach to model publication numbers from established and new literature databases. Humanit Soc Sci Commun. 2021 Oct 7;8(1):1–15.

6. Greene W. Functional forms for the negative binomial model for count data. Economics Letters. 2008 Jun 1;99(3):585–90.

7. Virtanen P, Gommers R, Oliphant TE, Haberland M, Reddy T, Cournapeau D, et al. SciPy 1.0: fundamental algorithms for scientific computing in Python. Nat Methods. 2020 Mar;17(3):261–72.

8. Seabold S, Perktold J. Statsmodels: Econometric and Statistical Modeling with Python. In Austin, Texas; 2010 [cited 2023 Mar 1]. p. 92–6. Available from: https://conference.scipy.org/proceedings/scipy2010/seabold.html

## Figure captions

**Figure E1**: Illustration of the study flow that started with a set of questions (yellow boxes), followed by data collection from PubMed (middle bottleneck) and a manual and automatic study (green boxes).

**Figure E2:** Flowchart with citation collection criteria used in the data collection process.

**Figure E3**: Cumulative count of publications related to causality and explainability.

**Figure E4:** Trend analysis. **A:** In the upper and lower portion of the figure, we displayed the goodness-of-fit measures SSE and $R^2$, respectively, which were obtained when varying the threshold date D for the indicator $\theta_D$ and fitting the piecewise NB1 Negative Binomial function from Equation 1. In the middle part, the piecewise fit corresponding to the best changepoint (October 2020). **B:** The Negative Binomial fit modelled by Equation 2. In light grey, the data points observed after October 2020; the corresponding points shifted by $\Delta$ months are shown in the right part of the plot. In both analyses, all parameters and their 95% confidence interval were estimated via bootstrapping (N=1000).

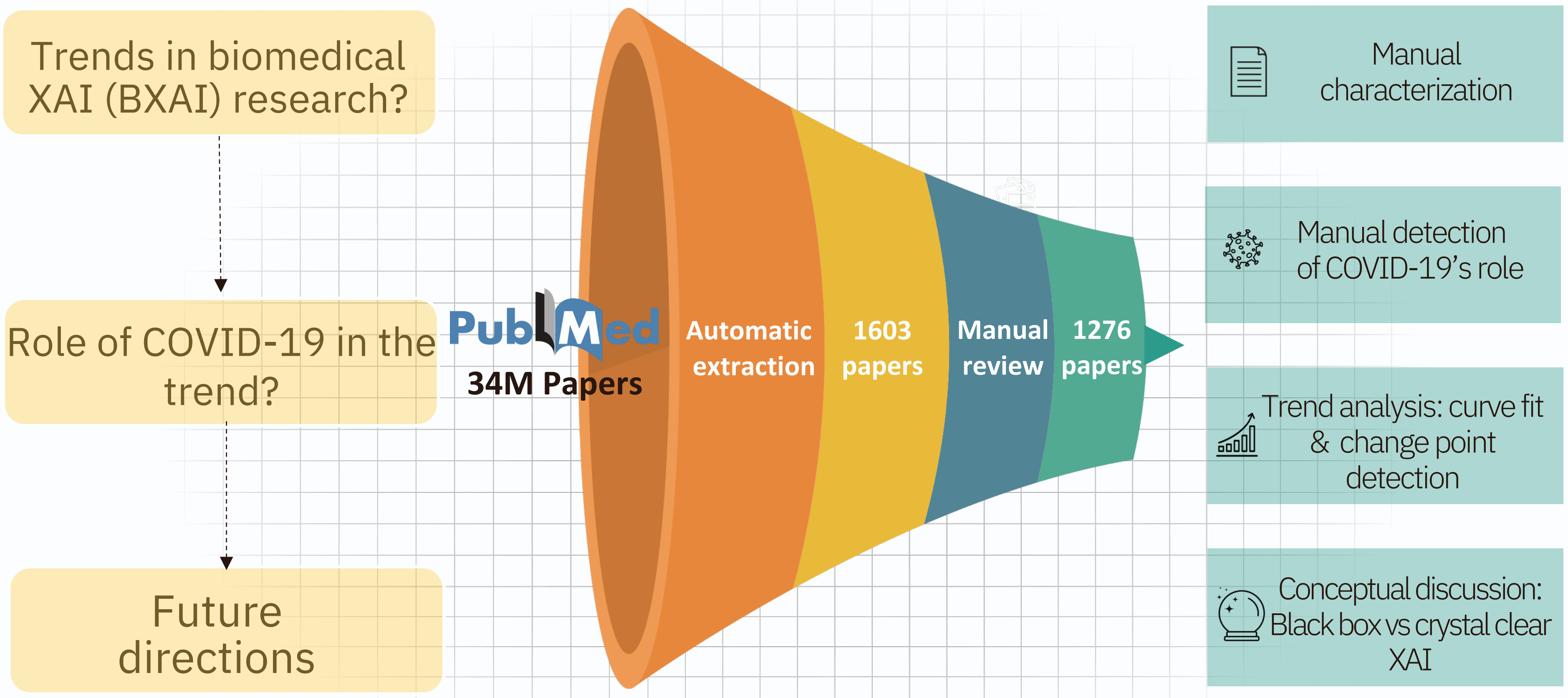
Trends in biomedical XAI (BXAI) research?
Role of COVID-19 in the trend?
Future directions
PubMed
34M Papers
Automatic extraction
1603 papers
Manual review
1276 papers
Manual characterization
Manual detection of COVID-19's role
Trend analysis: curve fit & change point detection
Conceptual discussion: Black box vs crystal clear XAI

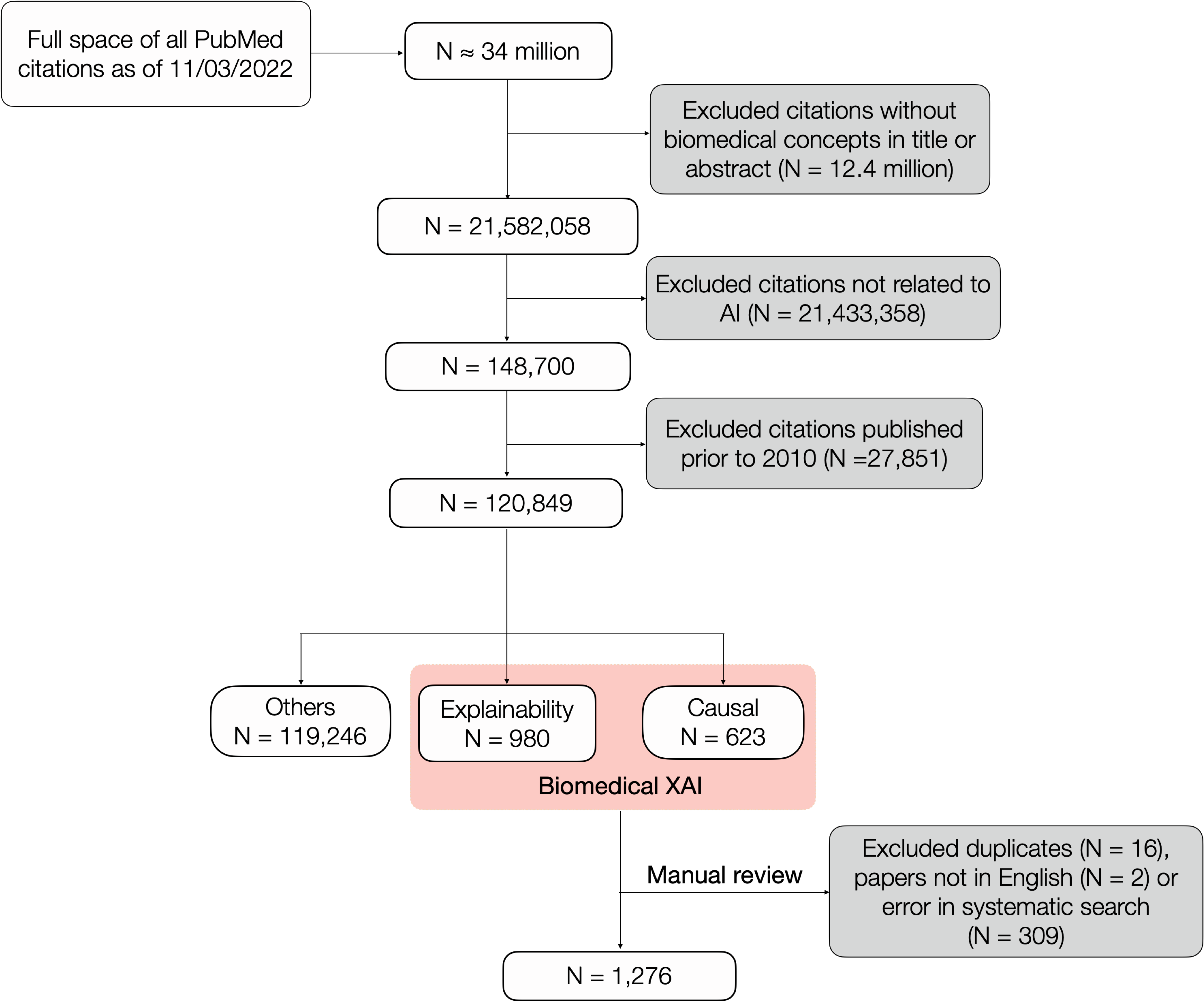
Full space of all PubMed citations as of 11/03/2022
N ≈ 34 million
Excluded citations without biomedical concepts in title or abstract (N = 12.4 million)
N = 21,582,058
Excluded citations not related to AI (N = 21,433,358)
N = 148,700
Excluded citations published prior to 2010 (N =27,851)
N = 120,849
Others N = 119,246
Explainability N = 980
Causal N = 623
Biomedical XAI
Manual review
Excluded duplicates (N = 16), papers not in English (N = 2) or error in systematic search (N = 309)
N = 1,276

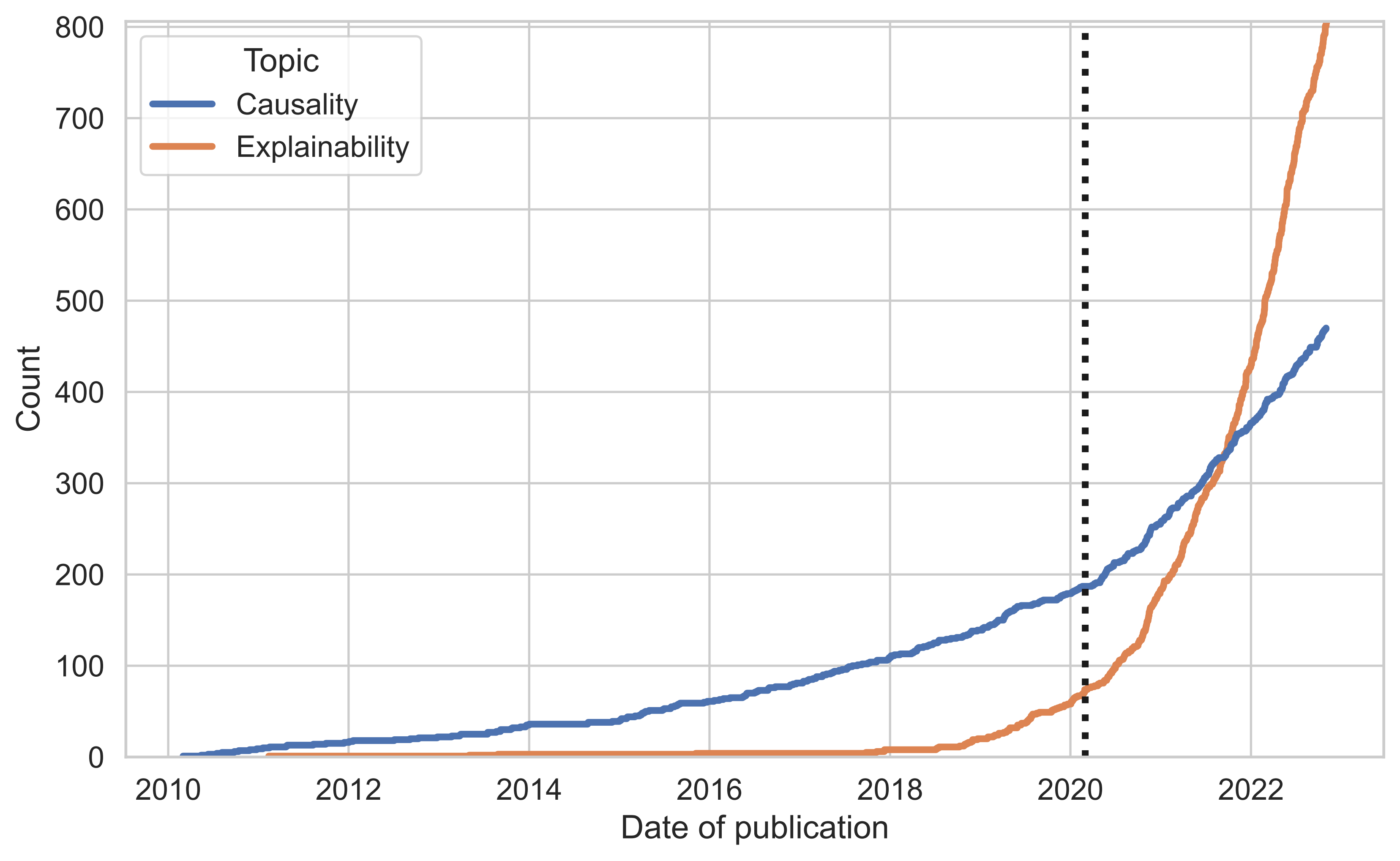
Topic
Causality
Explainability
Count
Date of publication
0
100
200
300
400
500
600
700
800
2010
2012
2014
2016
2018
2020
2022

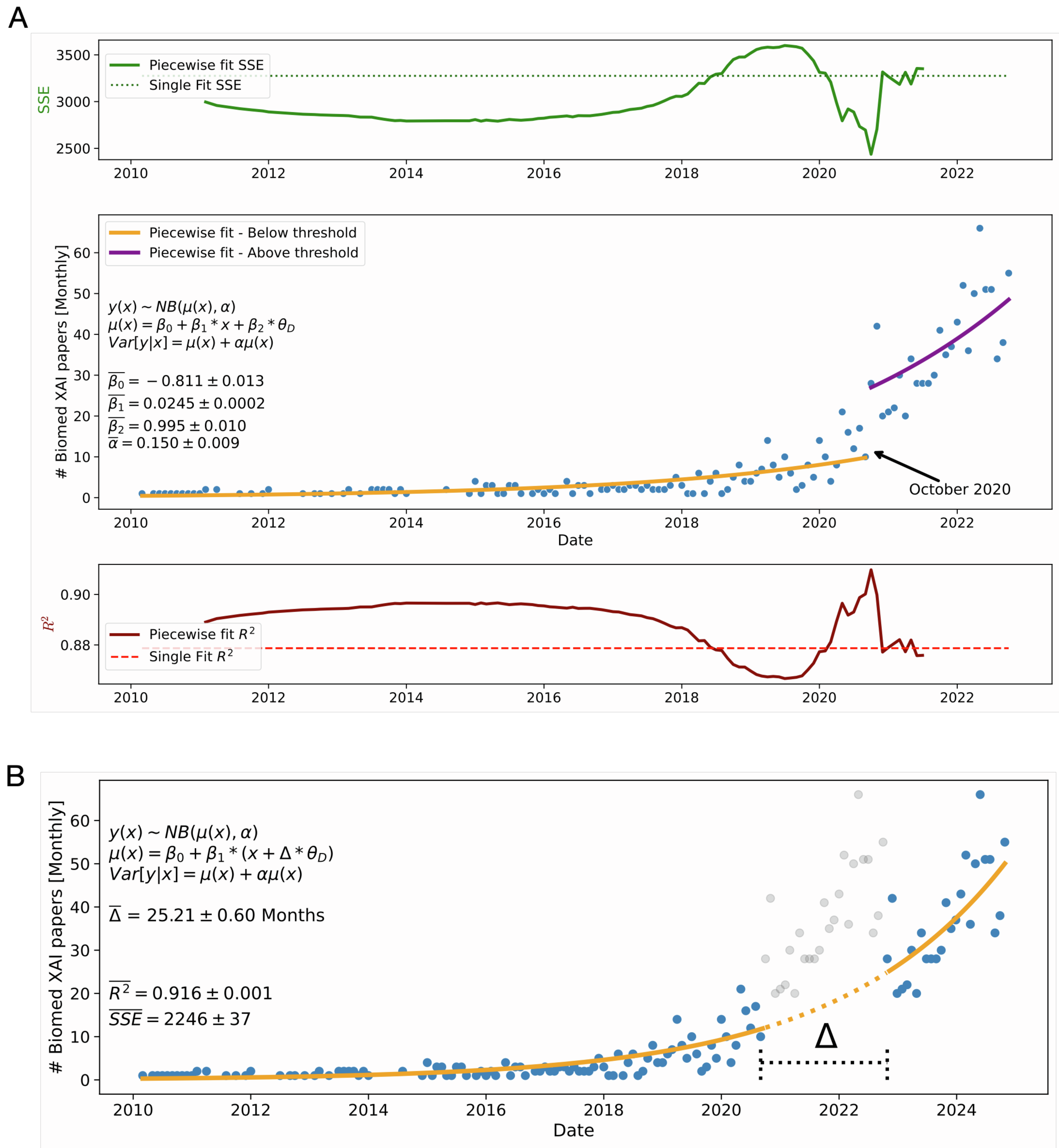
A
Piecewise fit SSE
Single Fit SSE
SSE
3500
3000
2500
2010
2012
2014
2016
2018
2020
2022
Piecewise fit - Below threshold
Piecewise fit - Above threshold
$y(x) \sim NB(\mu(x), \alpha)$
$\mu(x) = \beta_0 + \beta_1 * x + \beta_2 * \theta_D$
$Var[y|x] = \mu(x) + \alpha\mu(x)$
$\overline{\beta_0} = -0.811 \pm 0.013$
$\overline{\beta_1} = 0.0245 \pm 0.0002$
$\overline{\beta_2} = 0.995 \pm 0.010$
$\overline{\alpha} = 0.150 \pm 0.009$
October 2020
# Biomed XAI papers [Monthly]
60
50
40
30
20
10
0
Date
Piecewise fit $R^2$
Single Fit $R^2$
$R^2$
0.90
0.88
B
$y(x) \sim NB(\mu(x), \alpha)$
$\mu(x) = \beta_0 + \beta_1 * (x + \Delta * \theta_D)$
$Var[y|x] = \mu(x) + \alpha\mu(x)$
$\overline{\Delta} = 25.21 \pm 0.60$ Months
$\overline{R^2} = 0.916 \pm 0.001$
$\overline{SSE} = 2246 \pm 37$
$\Delta$
# Biomed XAI papers [Monthly]
2024
Date
